# Market competition and demand for skills in a credence goods market: Evidence from face-to-face and web-based non-physician clinician training in rural China

**DOI:** 10.1371/journal.pone.0233955

**Published:** 2020-06-18

**Authors:** Hongmei Yi, Paiou Wu, Xiaoyuan Zhang, Dirk E. Teuwen, Sean Sylvia

**Affiliations:** 1 China Center for Agricultural Policy, School of Advanced Agricultural Sciences, Peking University, Beijing, China; 2 Charles H. Dyson School of Applied Economics and Management, Cornell University, Ithaca, New York, United States of America; 3 Corporate Societal Responsibility, UCB, Brussels, Belgium; 4 Department of Health Policy and Management and the Carolina Population Center, University of North Carolina at Chapel Hill, Chapel Hill, North Carolina, United States of America; University of Jyvaskyla, FINLAND

## Abstract

**Background:**

Non-physician clinicians (NPCs) providing services in functionally private markets account for a large share of the workforce in the primary care system in many low-income and middle-income countries. Although regular in-service training is believed to be crucial to updating NPCs’ professional knowledge, skills, and practices, participation rates are often low. Low participation may result from the “credence good” nature of the market for primary care: if patients are unable to observe quality improvements from training, NPCs have weaker incentives to participate. Empirical evidence is limited on the relationship between market competition and NPC participation in-service training as well as how participation varies with the type of training available.

**Methods:**

The study uses a dataset of 301 NPCs from three prefectures in Yunnan, a province in southwest China, collected in July 2017. Logistic regression is used to estimate the relationship between competition and NPC’s participation in in-service training. We assess the relationship between participation and both the quantity of competition (number of competitors in the same village and surrounding villages) and the quality of competition (proxied using characteristics of competing clinicians).

**Results:**

In 2016, nearly two thirds of NPCs participated in face-to-face or web-based in-service trainings at least once. Specifically, 58 percent of NPCs participated in face-to-face in-service trainings, and 24 percent of NPCs participated in web-based in-service trainings. The quantity of competitors is unrelated to participation in in-service training. The quality of competition is not related to face-to-face training but has a significant positive relationship with participation in web-based training.

**Conclusions:**

Web-based trainings may be a better approach to increase NPC skills in developing country primary care markets.

## Introduction

Non-physician clinicians (NPCs) account for a large share of the workforce in the primary care system in many low-income and middle-income countries. The primary care system plays a fundamental role in many countries as the first point of contact with the health system [1, 2]. Due to shortages of physician clinicians, the primary care system in many low- and middle-income countries rely heavily on NPCs who have fewer clinical skills than physicians but more than nurses [3–8]. Daset al. [9] estimate that India has 1.6 million rural NPCs compared with just under 1 million clinicians with degree of bachelor of medicine and bachelor of surgery (MBBS), a large proportion of whom are concentrated in urban areas. In China, NPCs account for up to 62 percent of workforce in rural village clinics in 2017 [10]. Most sub-Saharan countries have scaled up training of NPCs, resulting in a gradual but decisive shift to NPCs as the cornerstone of healthcare delivery [11].

Although regular in-service training is believed to be crucial to updating NPCs’ professional knowledge, skills and practices, the participation rate is low [12]. NPCs usually have training in basic skills but little formal education and training in the fundamental medical sciences. As a result, repeated trainings are important to maintain and update NPC knowledge of appropriate patient care. A study in India has shown that training can effectively improve the quality of services provided by NPCs [9]. The World Health Organization (WHO) guidelines suggest that for NPCs to fulfil their role successfully, they require “regular training and supervision” [13]. However, existing evidence shows that only a small share of NPCs participates in the recommended amount of trainings, particularly when the training is not mandatory [14–16]. In China, for example, Liet al. [16] find that, although annual training is required by authorities, 36 percent of primary health-care clinicians, including NPCs, had not received the in-service training in the previous year.

This low rate of participation in trainings may result from the nature of the market for primary care. The primary care market is a typical credence goods market which are characterized by asymmetric information between sellers and consumers [17]. If quality is less observable to patients, there are reduced incentives for clinicians to provide high quality care. This has potential to result in a “market for lemons”, where the market degenerates until only low quality providers (i.e. quacks) are practicing medicine [18, 19]. In another words, in this market, clinicians have little incentive to improve their competence if they believe that quality is not observable to patients. Although, licensing requirements can potentially solve this problem by assuring a minimum level of competence in the market [19], such a system is often not viable in low- or middle-income countries given a lack of regulatory capacity. Licensing requirements may also exacerbate shortages of primary care, particularly in more sparsely populated rural areas.

On the other hand, if clinicians believe that quality is observable to patients, market mechanisms may provide sufficient incentives for NPCs to improve their quality of service. Competition has been found to reduce quality problems in credence goods markets in other contexts [20, 21]. For example, Mimraet al. [21] found that introducing costly second opinions in markets for expert services significantly reduces the level of overtreatment. Increased competition may therefore motivate NPCs to attend in-service trainings to update their skills and knowledge to improve the quality of services they provide, but only if they believe that they will gain skills that will allow them to credibly signal quality to patients and that patients respond to quality differences across providers.

Most existing studies on competition focused on the quantity of competition. A common practice is to use a measure of market intensity such as Herfindahl-Hirschman index to measure quantity of competition when examining the correlation between competition and hospital performance and patient outcomes [22–25]. When such information is not available, the number of neighboring providers has also been used to measure quantity of competition. For example, Daset al. [9] uses the presence of other providers in the same village as a proxy of competition faced by informal health care providers in India. Although few studies measure the quality of competition, there is strong evidence that clinicians’ age (or experiences) and education are good predictors of competitiveness of clinicians or patients’ choice of doctors [26–28].

Currently, there is also little evidence on the relationship between competition and clinicians’ participation in in-service training. Several descriptive studies suggest that direct costs (such as training fees), opportunity costs (loss of revenue during training period), transportation from clinics to training sites (for example, distance to training site, weather on the training day), and work load are the main factors that prevent rural clinicians from participating in face-to-face in-service trainings [9, 29–31]. Competition, however, may have different effects on different types of training. While theoretically, competition may increase clinicians’ demand for skills offered by training, clinicians faced with stronger competition may be reluctant to participate if participation requires time away from serving patients. In particular, because rural clinics are often far away from training locations and staffed with single clinicians, clinics may need to be closed when clinicians participate in face-to-face in-service training. In contrast, web-based trainings are flexible and accessible at different times during the day [32]. The opportunity cost in terms of lost patients may therefore be lower. As a result, the development of web-based training may change the relationship between competition and participation in in-service training. In general, to our knowledge, no existing empirical studies have examined the relationship between competition and NPC’s participation in in-service training or compared how this varies with the type of training available.

The goal of this study is to explore the relationship between competition and participation in in-service training of NPCs. To meet this goal, we use a dataset consisting of 301 NPCs in three prefectures in a southwestern province in rural China. We have three specific objectives: First, we document the history and nature of NPCs in rural China. Second, we describe the competition faced by NPCs and NPCs’ participation in two kinds of in-service training: face-to-face training and web-based training. Finally, we explore the correlation between competition, defined in terms of both quantity and quality of competition, and in-service training participation.

The rest of the paper is organized as follows. The second section introduces the history and nature of NPCs in rural China; section 3 presents data collection and the empirical approach; section 4 presents results; and we conclude and discuss implications in section 5.

## NPCs in rural primary care system in China

NPCs in China are primarily employed in village clinics serving rural areas. Village clinics are the frontline of rural primary care system and provide outpatient care services for common clinical conditions and public health services. Township health centers, the tier above village clinics, also provide outpatient care and public health services in addition to inpatient care services. Village clinics usually maintain a relationship with the township health centers above them, but function as independent for-profit entities with revenue generated from government subsidies, mainly through health insurance programs and basic public health services program [16]. China does not impose a strict referral or gatekeeping system so patients are free to where they seek initial primary care [12, 33, 34]. The majority of rural patients are first seen in village clinics particularly in remote areas [12, 33, 34].

Historically, China had one of the most successful models for training NPCs in the low- and middle-income countries. In the late 1960s, China initiated its “barefoot doctor” program to train non-physician clinicians to provide rudimentary care in rural areas. Most non-physician clinicians had completed junior high school education but lacked formal medical training. NPCs were able to practice in village clinics after training at county or community hospitals for 3–6 months. Studies have shown that this program significantly increased access to primary care for rural population and was held out as a model for other low- and middle-income countries [6].

After economic reform, China government made efforts to reinforce the workforce in village clinics to improve the quality of care accessible to the rural population. First, in 1985, non-physician clinicians began to be required to obtain a “village doctor” certificate to be permitted to practice in village clinics. This certification required an examination and those who failed were not permitted by local health authorities to practice in village clinics [35]. Second, since 1991, the government has issued a series of regulations on qualification and licensing of employees in village clinics, and guidelines for village clinicians’ education and training [36–42]. These policies have specified that village clinicians must have completed at least three-years of medical education after lower secondary education, have been practicing in village clinics for more than 20 years, or have completed required training by local governments [39]. Since 2001, new village clinicians are required to hold at least an `assistant physician’, a certification level above “village doctor” [37].

To date, most village clinicians are classified as NPCs. The health workforce in village clinics, based on their qualifications, are classified as certified physician clinicians, certified assistant physician clinicians, non-physician clinicians, and health workers [10]. Of the 1.45 million workforce in village clinics in 2017, 0.90 million (62%) were NPCs, 0.35 million (24%) were certified physician clinicians or certified assistant clinicians [10]. The proportion of NPCs is higher than this average in poor rural areas.

Although formally required by policy, evidence suggests that village clinicians participate in fewer in-service trainings than outlined by policy. According to national policy, village clinicians are required to receive at least two professional trainings every year, and annual accumulated training time should not be less than two weeks [43]. Studies have found, however, that many clinicians do not participate in the regular trainings or do not attend the amount of time required by the government [16, 30]. Face-to-face training is the most commonly used training method [12]. Meanwhile, web-based training has become a promising instructional method for reaching a large number of trainees [44]. More than 14 of China’s 34 province-level administrative units had adopted the web-based methods in rural clinicians’ training by 2009 [45]. In comparison with web-based training, face-to-face training is less accessible to trainees due to higher travel cost, less flexible timing, and limited training opportunities [32, 45].

China’s new healthcare reform, launched in March 2009, aims to provide safe, effective, convenient and affordable basic medical and health services to the entire population. A key aim of these reforms is to strengthen the quality of primary care including village clinicians. Although there are concerns about the quality of training available [29, 30], increasing village clinician participation in in-service trainings may be important to address the low quality of care that studies have found to be provided village clinicians [34, 46, 47]. Improving the quality of primary care in China’s rural areas is of particular policy concern given China’s plans to expand the scope of primary care and public health service provision to the rural population through village clinics [48–50].

## Methods

### Sampling

In this study, we use a dataset of NPCs collected in July 2017 from three prefectures in Yunnan, a province in southwest China. The per capita GDP in Yunnan province was 5068 US dollars in 2017, 42 percentage lower than the national level (8777 US dollars) [51]. There are 6.5 million rural residents in the three sample prefectures, accounting for 20 percent of Yunnan’s rural population [52].

The sampling procedure consisted of three steps. First, after excluding urban and minority counties in which minority population is more than 20 percent, we randomly selected a total of 10 rural counties in the three prefectures. Yunnan is a region with a high concentration of minority groups and has 25 minority nationalities. We chose non-minority prefectures to avoid complications with interviewing in minority languages. Second, we obtained a full list of village clinics and clinicians in these clinics from local health departments. We further excluded urban townships (containing the town center) and selected a total of 330 village clinics from 97 rural townships using probability proportional to size (PPS) sampling. Finally, when we visited the selected clinics in the field survey, we asked each clinic to list all employees serving in the clinic and describe their specialties and responsibilities. We excluded the 24% of health workers and clinicians who reported not practicing western medicine (i.e. those who only prescribe Chinese herbal medicine or are only responsible for public health services). We then randomly selected one village clinician from the list of each clinic as our sample.

In July 2017, we administered the survey to 330 village clinicians in the sample. Of 330 village clinicians, 301 (91%) were NPCs (with only a rural clinician certification) and 29 (9%) were physician clinicians or assistant physician clinicians. The distribution of clinics, clinicians, and NPCs among counties is presented in Table 1.

**Table 1:** Sample description.

County	Number of Townships	Number of Clinics	Number of Clinicians	Of which, Number of Non-Physician Clinicians (NPC) (%)
County 1	20	83	83	80(96.39%)
County 2	4	18	18	17(94.44%)
County 3	20	84	84	76(90.48%)
County 4	9	20	20	17(85.00%)
County 5	7	25	25	22(88.00%)
County 6	4	10	10	9(90.00%)
County 7	3	6	6	5(83.33%)
County 8	10	26	26	23(88.46%)
County 9	10	27	27	27(100.00%)
County 10	8	31	31	25(80.65%)
Total	95	330	330	301(91.21%)

### Data collection

The village clinician survey form consisted of four parts. First, we collected information on their demographics including age, sex, and ethnicity. Second, we asked about the highest-level of education completed, including formal and informal education, detailed schooling history including formal medical education, and use of internet services. In the third part, we asked a series of questions about village clinicians’ time allocation (full time or not, time spending on public health services, etc.) and income. The fourth part collected detailed information on village clinicians’ in-service training participation in 2016 (the year before the survey year). Specifically, we focused on county- or upper-level face-to-face trainings and web-based training. We asked each clinician whether he/she participated in any kind of in-service trainings. If yes, we further asked them a series questions to collect information on the organizer, duration, funding, and content for each piece of training. We also asked whether the clinic was closed or not during the training, and their subjective assessment on whether the training is helpful for NPCs to improve their skills.

The clinic facility survey included two parts. The first part collected basic information on clinics, including number of permanent residents within 5 kilometers of village clinic and distance from township health centers. In the seoncd part, we collect information on local wages of unskilled workers in the village.

In addition to clinician and clinic facility surveys, we obtained a full list of clinics and its clinicians in the sample counties from local health departments. The age and education for each clinician was also collected from local health departments. We use these variables to construct measures of competition.

### Measurement of competition

Because village clinics only provide outpatient services, the competition faced by NPCs mainly comes from other NPCs in other village clinics and township health centers. While we control for distance from village clinics to township health centers, we focus on measurement of competition from other NPCs, or their peers. Specifically, considering the fact that patients in China can freely choose health care providers, we define competitors of a clinician as clinicians who are in the same village or neighboring villages sharing the same boundaries.

We measure competition in terms of both quantity and quality. The measure of competition quantity was defined as the number of competitors in the same villages and surrounding villages. We measure the quality of competition as the education and age of clinicians in these same clinics–specifically, the share of competitors with higher education and share of competitors under 35 years old. The potential competitors for each clinic were determined using a list of all clinics and clinicians in each selected county obtained from local health departments (the same list used as our sampling frame). Competitors were identified as those clinics in the same village as the sample clinic and clinics in all villages sharing administrative boundaries with the village in which the sample clinic was located.

### Empirical strategies

We used the logistic regression to estimate the relationship between competition and NPC’s participation in in-service training. We conducted four types of regressions. In the first regression, we only include variables measuring the quantity of potential competitors. In the second regression, we only include variables measuring the quality of potential competitors. In the third regression, we include variables measuring both the quantity and quality of potential competitors. In the fourth regression, we further control for individual NPC characteristics, clinic characteristics, and village characteristics. All regressions control for prefecture fixed effects.

The specifications of these models are as follows:

$$Y_{ij}=\alpha +\beta_{1}{C1}_{ij}+\varphi D_{j}+\varepsilon_{ij}$$ (1)

$$Y_{ij}=\alpha +\beta_{2}{C2}_{ij}+\varphi D_{j}+\varepsilon_{ij}$$ (2)

$$Y_{ij}=\alpha +\beta_{1}{C1}_{ij}+\beta_{2}{C2}_{ij}+\varphi D_{j}+\varepsilon_{ij}$$ (3)

$$Y_{ij}=\alpha +\beta_{1}{C1}_{ij}+\beta_{2}{C2}_{ij}+\gamma X_{ij}+\delta {VC}_{ij}+\tau V_{ij}+\varphi D_{j}+\varepsilon_{ij}$$ (4)

where Y_ij_ is the outcome variables and represents whether NPC *i* from prefecture *j* attended any face-to-face or web-based in-service training in 2016 (combined), whether NPC *i* from prefecture *j* attended any face-to-face in-service training in 2016, or whether NPC *i* from prefecture *j* participated in any web-based in-service trainings in 2016. When we count the face-to-face training, only face-to-face trainings at county level or higher level are considered. C1_ij_ is a set of variables to measure the quantity of potential competition facing by NPC *i* from prefecture *j*. Specifically, C1_ij_ is number of competitors nearby (in the same village or neighboring villages). We use the log form of this variable because it is right-skewed. C2_ij_ is a set of variables to measure the quality of potential competition facing by NPC *i* from prefecture *j*. Specifically, C2_ij_ includes the share of competitors with higher education and the share of competitors under 35 years old.

X_ij_ is a vector of variables that measure NPC’s individual characteristics. This includes age, male, minority, education, any formal medical education, use of internet, full time or not, share of work time spending on public health services, and average daily income. VC_ij_ is a vector of variables that measure the characteristics of village clinics where NPCs are working. These controls include the number of permanent residents within 5 kilometers of village clinic and distance from township health centers. V_ij_ is daily wage of unskilled 50-year-old male worker which is an indicator of local economic development. D_j_ is a vector of prefecture dummies and ε_ij_ is a random error item.

### Ethics

Full ethical approval for this survey was obtained from the Peking University Institutional Review Board on April 26, 2017 (IRB00001052-17033). The board approved the verbal consent procedure. The verbal consent was obtained from local health departments and participants at the start of the survey without recording. We collected verbal consents for three reasons. First, the research presents no more than minimal risk of harm to subjects. Second, the research involves no procedures for which written consent is normally required outside of the research context. Finally, verbal consents are more culturally acceptable than written consents in the region we were working in.

## Results

### Characteristics of NPCs and competition

Table 2 presents the descriptive statistics of NPC, clinic and village characteristics. Of 301 NPCs, 65 percent are males and 11 percent are non-Han minorities. The average age of NPCs is 44.8 years old. Of which, 16 percent of NPCs are under 35 years old, and 10 percent of NPCs are older than 60 years old. The clinicians in the sample are younger than the national average. Most NPCs completed at least a high school education. However, among 301 NPCs, only 52 percent completed full-time formal medical education. Of these, 73 percent received full-time formal medical education in vocational high school and 27 percent received full-time formal medical education in college. More than 90 percent of NPCs use the internet.

**Table 2:** Characteristics of NPCs, clinics, and villages.

	Mean	Standard Deviation	Min	Max
**Number of observations**	301			
**NPC Individual Characteristics**				
Male (yes = 1)	0.65	0.48	0.00	1.00
Minority (yes = 1)	0.11	0.31	0.00	1.00
Age (years)	44.81	10.57	20.00	73.00
Education ^a^				
Junior high school or below (yes = 1)	0.07	0.26	0.00	1.00
Academic high school (yes = 1)	0.03	0.17	0.00	1.00
Vocational high school (yes = 1)	0.66	0.48	0.00	1.00
Higher education (yes = 1)	0.24	0.43	0.00	1.00
Full-time formal medical education ^b^ (yes = 1)	0.52	0.50	0.00	1.00
Use of Internet (yes = 1)	0.91	0.29	0.00	1.00
Full time or not (yes = 1)	0.54	0.50	0.00	1.00
Share of work time spending on public health service	0.56	0.23	0.00	0.99
Average daily income ^c^ (yuan)	80.51	48.16	6.58	365.53
**Clinic Characteristics**				
Number of permanent residents within 5 kilometers of village clinic	6453	9937	150	80000
Distance to township health center (km)	13.28	10.33	0.50	60
**Village Characteristics**				
Daily wage of unskilled 50-years-old male worker (yuan)	87.42	25.03	50	200

^a.^ Education includes formal education (receiving a certificate through full-time school education) and informal education (receiving a certificate through adult education, self-taught examination, correspondence courses, or trainings recognized only by local health departments).

^b.^ Full-time formal medical education includes education in upper secondary vocational high schools after junior high school and medical education in higher education system (for example, college, university). The certification through full-time formal medical education is recognized by both health departments and education departments across China.

^c.^ Average daily income includes income from both clinics and other sources such as farming and serving as a village cadre.

Table 3 shows the quantity and quality of potential competitors of NPCs. In terms of quantity of competitors, we find each NPC had an average of 10.46 competing clinicians nearby. Specifically, around 14 percent of NPCs had five or less competitors nearby, while 36 percent of NPCs had 6–10 competitors nearby, 37 percent of NPCs had 11–15 competitors nearby, and 12 percent of NPCs had 16 or more competitors nearby. In terms of the quality of competitors nearby, the average share of competitors with a higher education was 0.08 and the average share of competitors under 35 years old was 0.26.

**Table 3:** Competition characteristics.

Variables	Mean	Standard Deviation	Min	Max
**Quantity of potential competitors**				
Number of competitors nearby (number of clinicians)	10.46	4.73	1.00	31.00
Distribution of number of competitors (%)	100.00	--	--	--
≤5	14.29	--	--	--
6–10	36.21	--	--	--
11–15	37.21	--	--	--
≥16	12.29	--	--	--
**Quality of potential competitors**				
Share of competitors with a higher education	0.08	0.13	0.00	1.00
Share of competitors under 35 years old	0.26	0.19	0.00	1.00

### NPCs’ participation in in-service training

Table 4 reports provider participation in in-service trainings. In 2016, nearly two thirds of NPCs participated in face-to-face or web-based in-service trainings at least once. Specifically, 58 percent of NPCs participated in face-to-face in-service trainings, and 24 percent of NPCs participated in web-based in-service trainings.

**Table 4:** Characteristics of face-to-face and web-based in-service training.

Variables	Face-to-Face Training	Web-Based Training
**Number of clinicians**	301	
Participation in in-service training (Combined) (yes = 1)	0.66(200/301)	
Participation in face-to-face in-service training (yes = 1)	0.58(174/301)	
Participation in web-based in-service training (yes = 1)	0.24(71/301)	
**Number of trainings**	245	100
Free of charge (yes = 1)	1.00	0.83
Length of training (days)	10.16	3.52 ^a^
Clinic open during the training (yes = 1)	0.58	
Contents of training(yes = 1)		
Hypertension	0.67	0.67
Diabetes	0.64	0.65
Tuberculosis	0.54	0.42
AIDS	0.51	0.45
Mental health	0.51	0.43
Chronic lung diseases	0.36	0.31
Coronary heart diseases	0.30	0.32
Pediatric diarrhea	0.41	0.34
Rational use of antibiotics	0.54	0.46
Chinese herbal drugs	0.43	0.17
Traditional Chinese medicine physiotherapy	0.59	0.24
Physical examination	0.37	0.26
Emergency and first aid	0.46	0.35
Gynecological diseases	0.36	0.24
Male diseases	0.13	0.13
Orthopedic diseases	0.22	0.13
vSkin diseases	0.21	0.19
Surgical acute abdomen	0.27	0.23
Nursing	0.23	0.20
Rhinitis	0.16	0.13
Child epilepsy	0.12	0.07
Thyroid diseases	0.14	0.15
Others	0.24	0.28

^a.^ We calculate length of web-based training by multiplying the length of each course by number of courses, and then dividing by eight hours.

The face-to-face in-service trainings are different from web-based in-service trainings in cost, duration, and training content. We find that all of face-to-face in-service training are free while only around 83 percent of web-based in-service training are free. On average, a face-to-face training takes around 10.2 days while a web-based training takes 3.5 days. Each training usually covers more than one topic. Of a total of 245 pieces of face-to-face trainings, the most frequently mentioned topics are hypertension (67%), diabetes (64%), traditional Chinese Medicine physiotherapy (59%), rational use of antibiotics (54%), and tuberculosis (54%). Of a total of 100 pieces of web-based trainings, the most frequently mentioned topics were hypertension (67%), diabetes (65%), rational use of antibiotics (46%), AIDS (45%), and mental health (43%).

### Correlates between competition and NPCs’ participation in in-service training

Tables 5, 6 and 7 report the results of regressions relating competition measures to participation in face-to-face and web-based in-service training combined (Table 5), face-to-face in-service training (Table 6), and web-based in-service training (Table 7). We find that the quantity of potential competitors has no significant correlation with the participation in face-to-face and web-based trainings combined (Columns 1, 3, 4, Table 5) or in web-based in-service training (Column 1, 3, 4, Table 7). The relationship between quantity of potential competitors and participation in face-to-face in-service training are also insignificant when we don’t control for individual, clinic and village characteristic (Column 1, 3, Table 6). However, when we control for those variables, the coefficient is significantly negative at 10% significance level. The results indicate that every 1% increase in number of competitors nearby, the possibility of participating in face-to-face in-service training will be reduced by 0.10 percentage points (17%). Usually, face-to-face training resources are limited due to constraints such as human resources and spaces. When more competitors nearby compete for limited face-to-face training resources, NPCs will be less likely to have chance to participate even if they would like to do. However, web-based in-service trainings usually are less likely to have such constraints.

**Table 5:** Correlation between competition and NPCs’ participation in in-service trainings (combined).

Variables	(1)	(2)	(3)	(4)
**Competition Characteristics**				
Log(number of competitors nearby)	0.00		-0.02	-0.07
	(0.06)		(0.06)	(0.05)
Share of competitors with a higher education		0.77***	0.78***	0.50*
		(0.28)	(0.28)	(0.28)
Share of competitors under 35 years old		0.28*	0.28*	0.23
		(0.15)	(0.15)	(0.15)
**NPC Individual Characteristics**				
Male				0.16***
				(0.06)
Minority				-0.03
				(0.09)
Age				0.00
				(0.00)
Education				
Academic high school				0.01
				(0.18)
Vocational high school				0.13
				(0.10)
Higher education				0.18
				(0.12)
Full-time formal medical education				0.05
				(0.06)
Use of Internet				0.09
				(0.11)
Full time or not				0.09*
				(0.05)
Share of work time spending on public health				0.31***
Service				(0.12)
Log(average daily income)				0.10**
				(0.04)
**Clinic Characteristics**				
Log(number of permanent residents				-0.03
within 5 kilometers of village clinic)				(0.03)
Log(distance from township health center (km))				-0.02
				(0.04)
**Village Characteristics**				
Log(daily wage of unskilled 50-year-old male Worker)				0.10
				(0.10)
**Prefecture dummies**	YES	YES	YES	YES
Pseudo R-squared	0.03	0.06	0.06	0.15
LR test(chi-squared)	10.93	24.26	24.35	55.50
Joint significance of explanatory variables	0.01	<0.01	<0.01	<0.01
Observations	301	301	301	298

The table presents average marginal effects from logistic regressions, and standard error is in parentheses.

*** p<0.01,

** p<0.05,

* p<0.10.

**Table 6:** Correlation between competition and NPCs’ participation in face-to-face in-service trainings.

Variables	(1)	(2)	(3)	(4)
**Competition Characteristics**				
Log(number of competitors nearby)	-0.04		-0.05	-0.10*
	(0.06)		(0.06)	(0.06)
Share of competitors with a higher education		0.14	0.15	-0.12
		(0.23)	(0.24)	(0.23)
Share of competitors under 35 years old		0.25	0.26	0.23
		(0.16)	(0.16)	(0.16)
**NPC Individual Characteristics**				
Male				0.13**
				(0.06)
Minority				0.04
				(0.10)
Age				-0.00
				(0.00)
Education				
Academic high school				0.09
				(0.20)
Vocational high school				0.23*
				(0.12)
Higher education				0.13
				(0.14)
Full-time formal medical education				0.05
				(0.06)
Use of Internet				0.01
				(0.12)
Full time or not				0.09
				(0.06)
Share of work time spending on public health				0.23*
service				(0.13)
Log(average daily income)				0.12**
				(0.05)
**Clinic Characteristics**				
Log(number of permanent residents				-0.03
within 5 kilometers of village clinic)				(0.03)
Log(distance from township health center (km))				-0.03
				(0.04)
**Village Characteristics**				
Log(daily wage of unskilled 50-year-old male				0.09
Worker)				(0.10)
**Prefecture dummies**	YES	YES	YES	YES
Pseudo R-squared	0.01	0.02	0.02	0.09
LR test(chi-squared)	3.83	6.43	7.10	36.21
Joint significance of explanatory variables	0.28	0.17	0.21	0.01
Observations	301	301	301	298

The table presents average marginal effects from logistic regressions, and standard error is in parentheses.

** p<0.05,

* p<0.10.

**Table 7:** Correlation between competition and NPCs’ participation in web-based in-service trainings.

Variables	(1)	(2)	(3)	(4)
**Competition Characteristics**				
Log(number of competitors nearby)	0.05		0.04	0.01
	(0.04)		(0.04)	(0.04)
Share of competitors with a higher education		0.41**	0.39**	0.36**
		(0.16)	(0.16)	(0.17)
Share of competitors under 35 years old		0.35***	0.34***	0.36***
		(0.12)	(0.12)	(0.12)
**NPC Individual Characteristics**				
Male				0.04
				(0.05)
Minority				-0.05
				(0.08)
Age				0.00
				(0.00)
Education				
Academic high school				-0.18
				(0.18)
Vocational high school				-0.07
				(0.10)
Higher education				0.02
				(0.11)
Full-time formal medical education				-0.02
				(0.05)
Use of Internet				0.07
				(0.10)
Full time or not				0.01
				(0.05)
Share of work time spending on public health service				0.22**
				(0.12)
Log(average daily income)				-0.02
				(0.04)
**Clinic Characteristics**				
Log(number of permanent residents				0.01
within 5 kilometers of village clinic)				(0.03)
Log(distance from township health center (km))				-0.01
				(0.03)
**Village Characteristics**				
Log(daily wage of unskilled 50-year-old male				0.02
Worker)				(0.08)
**Prefecture dummies**	YES	YES	YES	YES
Pseudo R-squared	0.12	0.17	0.17	0.20
LR test(chi-squared)	39.67	54.83	55.73	64.06
Joint significance of explanatory variables	<0.01	<0.01	<0.01	<0.01
Observations	301	301	301	298

The table presents average marginal effects from logistic regressions, and standard error is in parentheses.

*** p<0.01,

** p<0.05.

In contrast to the quantity of competitors, we find that the proxies for quality of competitors are positively related to participation in combined in-service training. Specifically, we find that an increase of 1 (or 100 percentage points) in the share of competitors with higher education is associated with a 77 percentage point increase (an 117% increase) in training participation (Column 2, Table 5). Similarly, an increase of 1 (100 percentage points) in the share of competitors under 35 years of age is associated with a 28 percentage point (42%) increase in participation (Column 2, Table 5). The coefficients on these two variables remain significant after controlling for the quantity of competitors with little decline in the estimated relationship (Column 3, Table 5). When we further control for the individual, clinic and village characteristics, we find the coefficient on the share of competitors with higher education reduces to 50 percentage points (76%—significant at 10%) while the coefficient of the share of competitors under 35 years old is no longer significant (Column 4, Table 5).

The regressions by type of trainings suggest that the significant correlation between the quality of potential competitors and trainings are mainly driven by correlation with web-based in-service trainings. The results show that none of the quality competition variables is significantly related to attendance in face-to-face training in equation (Column2, 3, 4, Table 6). However, both quality competition variables are consistently correlated with web-based in-service training even after controlling for NPC and clinic characteristics (Column 2, 3, & 4, Table 7). These regressions suggest that an increase of 1 (100 percentage points) in the share of competitors with a higher education is related to an approximately 36–41 percentage point (150–171%) increase in web-based training participation. A similar relationship is found for the proportion of competitors under 35 years of age.

We also find that most other individual, clinic and village characteristics have little relationship with NPCs’ participation in any in-service training. When combining training types, we find that gender, full time or not, share of time spending on public health services and average daily income have a positive relationship with participation in in-service trainings (Column 4, Table 5).

In Tables 6 and 7, however we find that the factors associated with NPC’s participation in face-to-face trainings and web-based trainings vary. Specifically, the probability of attending face-to-face trainings for a male NPC is 13 percentage points (22%) higher than a female NPC. The probability of attending face-to-face trainings for a NPC with vocational high school education is 23 percentage points (40%) higher than a NPC with a junior high school education. Every 1% increase in average daily income also will increase the possibility of participating in face-to-face training by 12 percentage points (21%) (Column 4, Table 6). These variables are not related to participation in web-based trainings. However, Time spent on public health services is significantly related to both participation in face-to-face training and participation in web-based training (Column 4, Table 7). The positive correlation might be explained by that more trainings may be required for NPCs in order to helping them learn the skills for more public services.

## Conclusion with discussion

NPCs account for a large share of the workforce in the primary care system in many low-income and middle-income countries. China is no exception. As of 2017, 62 percent of village clinicians in rural village clinics were NPCs [10]. Although regular in-service training is believed to be crucial to updating NPCs’ professional knowledge, skills and practices, the nature of medical market might lead to low uptake of training.

In this study, we use a dataset of 301 NPCs from three prefectures in a province in southwestern China to examine the relationship between competition and NPCs’ participation in in-service trainings. We examine the relationship between participation in both face-to-face training and web-based training with the competition faced by NPCs in two dimensions: the quantity and quality of potential competitors.

We emphasize two key findings. First, the quantity of competitors has no significant relationship with participation in in-service trainings overall. Second, we find that, while the number of competitors is not associated with increased participation in face-to-face or web-based trainings, proxies for the *quality* of competition is significantly positively related to participation in web-based trainings. This latter result also suggests that clinicians believe that patient demand is driven at least in part by clinician skills and web-based trainings provide skills that allows them to compete with higher-quality competitors. Moreover, since these web-based trainings are often self-funded, they are willing to pay to acquire these skills. Taken together, the quality of competition plays a more significant role in improving in-service training, particularly web-based in-service training, than the quantity of competition.

In conclusion, this study provides new evidence that competition (specifically, the quality of competition) could drive primary care providers to improve their ability in credence goods markets. In particular, higher quality of potential competitors are more likely to push NPCs to invest in web-based trainings. Our analysis, however, only analyzed the correlation between competition and training participation. As such, the relationship we find may not be causal. Although we control for a number of observed factors that may confound the relationship, there may still be omitted variables that are correlated with both competition and training participation. For example, the priority of a local government to primary care may be correlated with more/higher quality competitors and encouragement of providers to participate in in-service training. In addition, although results suggest that the development of web-based trainings may be a viable way to increase NPC participation in regular trainings, the quality of web-based trainings and how this compares to face-to-face trainings, needs to be rigorously evaluated. Future studies are needed to explore the causal relationship between competition and training participation as well as the quality of trainings and impacts on provider performance.

## Supporting information

S1 File (PDF)

S2 File (PDF)
