# Supplementary material for: Market competition and demand for skills in a credence goods market: Evidence from face-to-face and web-based non-physician clinician training in rural China
Source: PLoS One. 2020 Jun 18;15(6):e0233955. doi: 10.1371/journal.pone.0233955 (PMC7302647; doi:10.1371/journal.pone.0233955)
Supplement: S1 File — (PDF) [file pone.0233955.s001.pdf]

问卷编码：□□□□□

2017年村诊所情况表1

市：________________

县（区）：________________

乡（镇）：________________

村：________________

村诊所名称：________________

负责人姓名：________________

负责人电话：__ __ __ __ __ __ __ __ __ __ __

被访者姓名：________________

被访者电话：__ __ __ __ __ __ __ __ __ __ __

访 谈　日 期：2017年____月____日　　　　访 谈 员 ：______________

访谈开始时间: ______时_______分　　　　访谈结束时间：______时______分

尊敬的医生：您好！

非常感谢您参加此次“云南农村卫生服务研究”问卷调研。该项目由中国红十字会总会事业发展中心和优时比共同设立的“健康希望基金”资助，委托北京大学开展，旨在通过了解农村基层医疗卫生机构现状，为进一步改善农村医疗卫生条件提供政策建议。您的支持和配合对我们顺利完成调研工作至关重要，希望您根据真实情况准确填答，不要遗漏。我们承诺答卷内容仅用于研究，并严格保密，请不要有任何顾虑。再次感谢您的合作！

调研员须知：

1）调研员需要对受访医生读完每个问题和选项。请将医生回答的答案写在答案列。若无特殊说明，均指2017年（今年）情况。

2）若要求填写数值，请填写具体数值，而非区间。如果医生刚开始习惯说XX-XX区间，给医生说“我们每次都需要一个具体的数字，而不是XX到XX。请您尽可能给我们一个具体的数字。”提醒3次后，如果医生仍然没有给出具体数值，调查员取区间的平均值。

3）如果村医不知道如何回答时，填888，并且文字备注医生不知道如何回答的原因；如果村医有特殊情况，调查员不知道如何填写，填999，并且文字备注特殊情况具体是指什么。

4）题目中括号内的部分不需要读出来，等医生有问题涉及到了再告诉医生。

A.村诊所工作人员名单

指导语：下面将向您了解村诊所的人员情况

a．该村诊所工作人员姓名		b．性别<br>1=男<br>2=女	c．年龄（岁）	d．在本诊所的职责<br>1=负责问诊和开处方的医生<br>2=负责问诊和开处方，但只开中草药的医生>>下一人<br>3=不负责问诊和开处方的医生》》下一人	e.是否做医生表？（若该医生在该诊所工作的时间从现在算起不超过4个月，则不需要）<br>1=是➔下一人<br>2=否	备注（未调查的原因）
1.						
2.						
3.						
4.						
5.						

B.村诊所的基本情况

<u>指导语：</u>下面将向您了解一些村诊所的基本情况

问　　　　题		单位/选项	答案
1.	您这个诊所负责问诊和开处方的医务人员有几个人是乡镇卫生院的正式职工？（这一题的人数应该小于或等于表 A 中第 d 题选择 1 的医生人数之和）	人 **>>填 0，跳第 3 题**	
2.	有乡镇卫生院正式编制的人员姓名		
3.	您这个诊所是否是新农合的定点医疗机构？	1=是<br>2=否>>C 部分	
4.	本村参合村民在您这个诊所看病的花费能否从新农合报销？	1=能　　2=不能	

C.服务

C1.基本情况

<u>指导语：</u>下面将向您了解一些诊所提供的服务的基本情况。

问	题	单位/选项	答案
1.	您诊所周边方圆 5 公里内，包括您这个诊所一共有多少家诊所，只要能看病的诊所都算？	家	
2.	您诊所周边方圆 5 公里内有多少家药店，卖药的小卖部也算，不包括诊所？（没有请填 0）	家	
3.	您诊所周边方圆 5 公里内，大概有多少常住人口，本地居民和外来人口中的常住人口都算？	人	
4.	您诊所周边方圆 5 公里内，平均有百分之多少的常住人口来您这里看病？	%	
5.	上个月，您这个诊所一共给多少人次的病人看病？	人次	
6.	其中，您建议了多少人次的病人转诊到上级医院？（6<5）	人次	
7.	上个月，有多少个您不认识的病人来您这里看病？	人	
8.	您这个诊所是否收取一般诊疗费，对参合患者减免的也算收？	1=是，针对所有人都收<br>2=是，针对部分人收（具体请说明）<br>3=否 <u>>>10 题</u>	
9.	如果收，一般诊疗费是多少钱？(需要核实医生说的诊疗费是否是减免后的)	元/人次	
10.	您这个诊所是否打肌肉针或者打小针？	1=是　　2=否 <u>>>12 题</u>	
11.	上个月，来打肌肉针的有多少人次？(11<5)	人次	
12.	您这个诊所是否为病人进行静脉输液，打吊瓶？	1=是　　2=否 <u>>>14 题</u>	
13.	上个月，到您这个诊所来进行静脉输液，打吊瓶的有多少人次？(13<5)	人次	
14.	2016 年，您这个诊所是否承担公共卫生服务？	1=是　　2=否 <u>>>C2 部分</u>	
15.	2016 年,您这个诊所承担多少个行政村的公共卫生服务？	个（可填小数，例如 0.5）	
16.	2016 年，上述行政村共多少人？	人	

C2.医疗设备配备及使用

指导语：下面将向您了解医疗设备的配备及使用情况。 我问到具体设备的时候，麻烦您带我去看一下。

调研员须知：注意所有设备要眼见为实，调查员需和医生一起确认有该设备。如果某一设备坏了并且医生不打算再修，则算没有。

设备名称	a．是否有该设备？<br>1=有<br>2=没有>>下一行	b．现在用的是否是上级机构免费配发的？<br>1=是<br>2=否	c．上周是否用过该设备？<br>1=是<br>2=否
1. 听诊器			
2. 体温计			
3. 血压计			
4. 压舌板			
5. 止血带			
6. 治疗盘			
7. 出诊箱			
8. 带压力表的高温消毒锅			
9. 紫外线消毒灯			
10. 清创缝合包			
11. 病床			
12. 吸痰器			
13. 冷藏包或者冷藏袋			
14. 氧气包或者氧气袋或者氧气瓶			
15. 血常规化验仪			
16. 血糖监测仪			
17. 身高体重计			
18. 带盖垃圾桶			
19. 电脑			
20. 打印机			
21. 药柜			
22. 真空拔罐器			
23. 经穴治疗仪			
24. 艾灸盒			
25. 按摩床			
26. 多参数健康检测一体机			
27. 冰柜或冰箱			
28. 特定电磁波治疗仪或者神灯			
29. 其他 1，请说明 ______			
30. 其他 2_，请说明____			
31. 其他 3_，请说明____			
32. 其他 4_，请说明____			

33. 其他 5_，请说明____			
34. 其他 6_，请说明____			
35. 其他 7_，请说明____			
36. 其他 8_，请说明____			

D. 就诊记录

指导语：下面了解贵诊所就诊记录的情况，麻烦您把和病人就诊记录相关的资料都拿出来，我们一起看着更方便我们继续访谈。

调研员须知：对于1-11题，问医生有哪些记录和病人看病相关信息的资料，并且让医生全部拿出来。然后根据所有类型的记录回答相关问题；村医如果只保存少数特殊病人的记录（例如，只保存欠账病人的就诊记录）不算保存就诊记录。

问　　题		单位/选项	答案
1.	您这个诊所保存病人就诊记录，例如，病人个人信息、门诊日志、处方等信息吗？（如果就诊记录仅仅是记录患者的姓名和就诊日期，则不算就诊记录，该题应选择 2=否）	1=是　2=否　**>>8 题**	
2.	就诊记录主要以什么形式保存？	1=纸质版<br>2=电子版<br>3=纸质版和电子版都有	
3.	就诊记录是否包括患者的个人信息(姓名、性别、年龄等)？	1=是　2=否	
4.	就诊记录是否包括患者的主要症状？	1=是　2=否	
5.	就诊记录是否包括患者的既往病史？	1=是　2=否	
6.	就诊记录是否包括对患者的诊断结果？	1=是　2=否	
7.	就诊记录是否包括对患者的治疗方案（处方）？	1=是　2=否	
8.	2016 年，上级机构是否对村诊所保存的病历或处方资料进行检查？	1=是　2=否　**>>E 部分**	
9.	2016 年，多长时间检查一次？	1=三个月内<br>2=三个月到六个月内<br>3=六个月到九个月内<br>4=九个月到一年内<br>5=一年以上	
10.	检查时如果发现有明显的错误，会进行罚款吗？	1=会　2=不会	
11.	检查时如果发现有明显的错误，会进行口头批评、通报批评等行政处罚吗？	1=会　2=不会**>>E 部分**	
12.	检查时如果发现有明显的错误，会进行哪些行政处罚吗？（可多选，读完所有选项。）	1=口头批评<br>2=通报批评<br>3=吊销村医执业证书<br>4=吊销村诊所营业执照<br>5=其他，请说明	

E. 药品配备与使用

<u>指导语</u>：下面了解诊所药品的配备及使用情况。

问　　题		单位/选项	答案
1.	2016 年年底时，贵诊所库存**西药品种数**	品种数	
2.	2016 年年底时，贵诊所库存**中成药品种数**	品种数	
3.	2016 年年底时，贵诊所库存**中草药品种数**	品种数	
4.	2016 年年底时，贵诊所库存药品的总价值是多少？	元	
5.	2016 年年底时，按照**采购药品品种**算，有百分之多少的药品是您自己进/采购的，不包括从国家基本医药平台、乡镇卫生院采购的药品？	%（没有填 0）	
6.	2016 年年底时，按照**采购金额**算，有百分之多少的药品是您自己进/采购的，不包括从国家基本医药平台、乡镇卫生院采购的药品？	%（如果第 5 题是 0，则该题为 0）	
7.	贵诊所是否执行药品零差价政策？（即药品销售价与进价相同）	1=是<br>2=否<u>>>**F 部分**</u>	
8.	2016 年，上级机构是否对贵诊所药品零差率执行情况进行考核？	1=是<br>2=否	

F. 村诊所的地理位置

<u>指导语</u>：最后，想了解本村的地理位置信息及经济情况？

问　　题		单位/选项	答案
1.	这个诊所距离乡镇卫生院有多远？	公里	
2.	每天有没有从本村去乡镇卫生院的班车或面包车？	1=有<br>2=没有<u>>>第 4 题</u>	
3.	病人从您村到最常去的乡镇卫生院，如果坐班车或面包车单程大概需要多少钱，租私人车不算？（取最高价）	元	
4.	这个诊所距离村民最常去的县医院有多远？	公里	
5.	每天有没有从本村或者乡镇到村民最常去的县医院的班车或面包车？	1=有<br>2=没有<u>>>第 7 题</u>	
6.	病人从您村到村民最常去的县医院，如果坐班车或面包车单程大概需要多少钱，租私人车不算？（取最高价）	元	
7.	您村 50 岁左右的男性在本村打零工（不是技术工），一天的工资平均多少钱？	元	
8.	您村 50 岁左右的女性在本村打零工（不是技术工），一天的工资平均多少钱？	元	

填表人（签名）___________

查表人（签名）___________

问卷编码：□□□□□

2017 年村诊所情况表 2

A.常见病例管理情况

指导语：接下来，我们继续了解**您这个诊所**对常 w 见病例的管理情况。

调研员须知：按列进行提问，将每个病例完整代入问题给医生读。

	肺结核	II 型糖尿病	高血压	咽炎	小儿腹泻	哮喘	慢性肺病	心绞痛
1.2016 年，您这个诊所是否负责 XX 病人的管理？（1=是 2=否>>3 题）				☒	☒	☒	☒	☒
2.2016 年，您这个诊所一共管理了多少 XX 的病人？（如果没有，填 0）				☒	☒	☒	☒	☒
3.2016 年，您这个诊所新发现了多少疑似 XX 的病人？【如果为 0，跳至下一病例】								
4.其中,您建议转诊(包括口头转诊）了多少个疑似 XX 的病人？无论病人去不去都算。								
5.其中，您给上级机构正式报备了多少个疑似 XX 的病人？		☒	☒	☒	☒	☒	☒	☒

B.2016 年的收入

指导语：下面将向您了解一些 2016 年的收入情况。我们一起来算一算。

调研员须知：若诊所情况表 1 的 E 部分第 5 和 6 题的村医自己采购药品部分不为零，则一定会有“非零差率药品纯收入”。（如果有雇佣的医生，第 7 题应该要包括该医生的工资，算作是村诊所的纯收入，这样才能让该医生来分配诊所总收入的一部分。）

问	题	单位/选项	答案
1.	进行基本公共卫生服务获得的补助	元	
2.	因实行药品零差率获得的补助	元	
3.	诊所所有人员从县卫生局得到的年固定工资	元	
4.	医疗纯收入，不包括药品收入	元	
5.	非零差率药品纯收入	元	
6.	其他收入（如果是村医自己种地的收入或其他与诊所无关的收入不算），请说明____	元	
7.	村诊所纯收入总计（要先问医生纯收入总和，然后再将该数字与 1 题至 6 题金额加总进行对比，如有出入，确认原因；7=1+2+3+4+5+6）	元	

问卷编码：□□□□□□

2017 年村诊所医务人员表

市：________________________________

县（区）：________________________________

乡（镇）：_________________________________

村：_________________________________

村诊所名称：_________________________________

村医姓名：_________________________________

村医电话：__ __ __ __ __ __ __ __ __ __ __

访 谈 日 期：2017 年_____月____日_ 访 谈 员 ：_______________

访谈开始时间: _______时_______分 访谈结束时间：_______时_______分

指导语：接下来，我们开始做医务人员表，主要了解您诊所所有负责问诊和处方的医生（诊所表中A部分第e题回答“1”的医生总数）的基本情况。

A. 医生基本信息

A1. 个人信息

指导语: 首先是关于您个人基本信息的一些问题

问 题		单位/选项	答 案
1.	性别	1=男　　2=女	
2.	您的民族	1=汉族　　2=彝族<br>3=白族　　4=哈尼族<br>5=壮族　　6=苗族<br>7=回族　　8=傈僳族<br>9=拉祜族　　10=佤族<br>11=纳西族　　12=瑶族<br>13=其他，请说明_______	
3.	出生年月日	例：19670120	
4.	您是否是本村人？	1=是　　2=否	
5.	您在贵诊所主要负责什么？	1=全部工作<br>2=医疗<br>3=公共卫生<br>4=其他，请说明	
6.	您最早从哪一年开始当医生的？	年份，例如 1988	
7.	您中途不做医生，而是从事其他职业的时间有多少年（兼职做其他工作不算）？	年（如果未从事过其他职业，填 0；可以填小数）	
8.	从哪一年起，您开始在现在这个诊所工作？（注意年份不要早于出生年份）	年份，例如 1997	
9.	您当医生的最主要原因是什么?（必须读完所有选项）	1=亲戚或家人是医生<br>2=以前学的是医学<br>3=当时村里没有村医<br>4=喜欢当医生<br>5=当医生赚钱养家<br>6=当时村集体选举的<br>7=其他，请说明	
10.	您在从医之前，您的家人或亲戚是否是村医？	1=是　　2=否	
11.	您是否希望您的孩子或者孙子女以后当村医？	1=是　　2=否	
12.	2016 年，在与医生相关的工作时间内，您用于公共卫生服务的时间占了百分之多少？（小于 100）	%	
13.	上周，您做医生相关的工作共用了多少小时？要扣除吃饭、睡觉、休闲以及做其他事情的时间。	小时（小于 168 小时）	

A2.学历

	问 题	单位/选项	答 案
1.	包括成人教育在内，您现在已获得的最高学历是什么（请医生拿出证书进行核对）？	0=无<br>1=小学及以下（包括初中未毕业）<br>2=初中毕业（包括高中未毕业）<br>3=高中毕业<br>4=中职（中专、技校、职高）毕业<br>5=大专 毕业<br>6=本科毕业<br>7=研究生及以上毕业	
2.	您现在已获得的最高执业资格证书是什么？（请医生拿出证书进行核对）	0=没有<br>1=乡村医生资格证书<br>2=执业助理医师<br>3=执业医师<br>4=其他，请注明	
3.	您是否接受过任何**有学历证书**的医学教育，包括全日制脱产学习、在职教育、函授等？（请医生拿出证书进行核对）	1=是<br>2=否 >>9 题	

指导语：接下来我们想了解一下您每一次接受的医学学历教育的情况，这里的医学学历教育包括全日制脱产学习、在职教育、函授等拿到学历证书的教育，但是不包括培训。**【最好能看到医生的学历证书确认一下是否是学历教育】**
调查员须知：第 4 至第 8 题需要针对第 3 题的结果，并完整陈述每道题的问题。

	问 题	单位/选项	医学教育			
			第一次	第二次	第三次	第四次
4.	获得这次学历的年份	年（例如：1990）				
5.	是否是全日制脱产学习，也就是完全在学校学习不工作？	1=是 2=否				
6.	这次获得的是什么学历？<br>**（注意：如果医生不能确认，需要调查员根据学历证书确定是哪个等级）**	1=中职（中专、技校、职高）<br>2=大专<br>3=本科<br>4=研究生及以上				
7.	这次学历教育学的是什么专业？	1=中医<br>2=西医<br>3=中西医结合<br>4=公共卫生<br>5=护理学<br>6=无具体专业<br>7=其他，请注明				
8.	这次学费是否全部由您自己支付？	1=是 2=否				

9.	您在当医生之前，是否接受过任何的非学历性的医学训练？	1=是 2=否 >>A3 部分	
10.	您在当医生之前，一共接受了多长时间的非学历性的医学训练？	天	

A3. 培训

指导语：接下来想了解您2016年参加过的面对面的医疗培训的具体情况。

	问题	选项	答案
1.	2016 年您是否参加过乡镇卫生院的医疗培训？	1=是<br>2=否 >>4 题	
2.	如果参加过，2016 年参加过几次乡镇卫生院组织的培训？	次	
3.	2016 年，乡镇卫生院培训的主要内容是什么？	1=医疗为主<br>2=公共卫生为主<br>3=医疗和公共卫生并重<br>4=其他，请说明	
4.	2016 年，除了乡镇卫生院的培训，您是否参加过其他任何面对面的医疗培训（不包括远程或在线培训）	1=是<br>2=否 >>23 题	

	问题	选项	答案				
			a. 县卫生局	b. 市卫生局	c. 其他机构 1:_______	d. 其他机构 2: _______	e. 其他机构 3:______
5.	2016 年您是否参加过 XXX（PAD 上，依次问“县卫生局”、“市卫生局”、“其他机构”）组织的医疗培训？	1=是<br>2=否 >>**下一列**					
6.	如果参加过，2016 年参加过几次该机构组织的培训？	次					
7.	这是该机构组织第几次的培训？	次	县卫生局第 X 次	市卫生局第 X 次	其他机构 1:_______ 第 X 次	其他机构 2: _____ 第 X 次	其他机构 3:______ 第 X 次
8.	本次培训是在几月份开始的？	月份（例如:7）					
9.	本次培训一共多长时间，不包括路上往返时间	天（可以填小数）					
9.1.	实际参加的培训一共多长时间，不包括路上往返时间(9.1<=9)	天（可以填小数）					

10.	本次培训是否有临床实践？	1=有 2=没有						
11.	本次培训的课程是否免费？	1=是 >>13 题<br>2=否						
12.	如果不免费，培训费多少钱？	元						
13.	本次培训产生的交通食宿费用是否**主要**由您自己承担？	1=是<br>2=否 >>15 题						
14.	如果由您承担，交通食宿费一共多少钱？	元						
15.	本次培训期间，您的诊所是否正常营业？	1=是>>17 题<br>2=否						
16.	假如您的诊所正常营业，培训这段时间内您估计能赚多少钱？	元						
17.	培训后一个月内，您向培训专家咨询的次数？	次（没有请填 0）						
18.	您觉得本次培训对提高村医医疗技能是否有帮助？	1=完全没有帮助<br>2=基本没有帮助<br>3=有一些帮助<br>4=比较有帮助<br>5=非常有帮助						
19.	如果需要付费，您愿意为本次培训最多支付多少元培训费用？	元						
20.	如果需要付费，您愿意为本次培训最多支付多少交通食宿费用？	元						
21.	本次培训的讲课老师主要来自哪里？	1=村级/社区的医生<br>2=乡镇/街道办级别医生<br>3=县级的医生<br>4=地州级的医生<br>5=省级及以上医院<br>6=其他，请说明						
22.	本次培训讲到了哪些内容？							
22.1	高血压	1=是 2=否						
22.2	糖尿病	1=是 2=否						
22.3	肺结核	1=是 2=否						

22.4	艾滋病	1=是　　2=否					
22.5	精神障碍或精神病	1=是　　2=否					
22.6	慢性肺病	1=是　　2=否					
22.7	冠心病	1=是　　2=否					
22.8	小儿腹泻	1=是　　2=否					
22.9	抗生素的合理使用	1=是　　2=否					
22.10	中草药	1=是　　2=否					
22.11	中医理疗	1=是　　2=否					
22.12	体格检查	1=是　　2=否					
22.13	急诊与急救	1=是　　2=否					
22.14	妇科疾病	1=是　　2=否					
22.15	男科疾病	1=是　　2=否					
22.16	骨科疾病（如骨折与骨关节炎）	1=是　　2=否					
22.17	皮肤病	1=是　　2=否					
22.18	外科急腹症	1=是　　2=否					
22.19	护理	1=是　　2=否					
22.20	鼻炎	1=是　　2=否					
22.21	儿童癫痫	1=是　　2=否					
22.22	甲状腺类疾病	1=是　　2=否					
22.23	其他，请说明__________	1=是　　2=否					
22.24	其他，请说明__________	1=是　　2=否					
22.25	其他，请说明__________	1=是　　2=否					
22.26	其他，请说明__________	1=是　　2=否					
22.27	其他，请说明__________	1=是　　2=否					
22.28	其他，请说明__________	1=是　　2=否					

问 题		单位/选项		答 案
23.	如果有机会，您最想接受哪方面的培训（不包括远程或在线培训）？<br>**（单选）(必须让医生知晓所有选项）**	0=什么培训都不想接受>>**28 题**<br>1=高血压<br>2=糖尿病<br>3=肺结核<br>4=艾滋病<br>5=精神障碍或精神病<br>6=慢性肺病<br>7=冠心病<br>8=小儿腹泻<br>9=抗生素的合理使用<br>10=中草药<br>11=中医理疗<br>12=体格检查	13=急诊与急救<br>14=妇科疾病<br>15=男科疾病<br>16=骨科疾病（如骨折与骨关节炎）<br>17=皮肤病<br>18=外科急腹症<br>19=护理<br>20=鼻炎<br>21=儿童癫痫<br>22=甲状腺疾病<br>23=其他，请说明	
24.	对于最希望接受的培训，您最希望以哪种方式进行？<br>**（单选）（不包括远程或在线培训）**	1=专题讲座<br>2=互动教学<br>3=情景模拟法<br>4=临床实践<br>5=其他，请说明		
25.	对于最希望接受的培训，您最长愿意脱产参加多少天？	天（可以填小数）		
26.	对于最希望接受的培训，您愿意最多支付多少培训费用，仅指培训费用，不包括交通食宿费用？	元		
27.	对于最希望接受的培训，您是否愿意自己承担交通食宿费用？	1=是 2=否		
28.	2013-2015 年这三年内，您是否参加过县级及以上的培训？	1=是 2=否		
29.	2013-2016 年这四年内，您是否在上级医院进修实习过（不包括之前提到的培训）？	1=是 2=否		

远程培训

指导语：接下来想了解您2016年参加过的远程在线培训的具体情况。

问 题		单位/选项	答 案
30.	2016 年，您是否参与过任何形式的和村诊所工作相关的远程在线培训？	1=是 2=否 >>40 题	
31.	如果参加过，参加过几次？	次	

			第一次	第二次	第三次	第四次
32.	您是从什么渠道获得这次培训的机会？	1=上级机构 2=其他医生<br>3=互联网 4=其他，请说明				
33.	您是在哪里参加的这次培训？	1=自己家里/诊所 2=村委会<br>3=乡镇卫生院 4=其他，请说明				
34.	本次培训讲到了哪些内容？					
34.1	高血压	1=是 2=否				
34.2	糖尿病	1=是 2=否				
34.3	肺结核	1=是 2=否				
34.4	艾滋病	1=是 2=否				
34.5	精神障碍或精神病	1=是 2=否				
34.6	慢性肺病	1=是 2=否				
34.7	冠心病	1=是 2=否				
34.8	小儿腹泻	1=是 2=否				
34.9	抗生素的合理使用	1=是 2=否				
34.10	中草药	1=是 2=否				
34.11	中医理疗	1=是 2=否				
34.12	体格检查	1=是 2=否				
34.13	急诊与急救	1=是 2=否				
34.14	妇科疾病	1=是 2=否				
34.15	男科疾病	1=是 2=否				
34.16	骨科疾病（如骨折与骨关节炎）	1=是 2=否				
34.17	皮肤病	1=是 2=否				
34.18	外科急腹症	1=是 2=否				
34.19	护理	1=是 2=否				
34.20	鼻炎	1=是 2=否				
34.21	儿童癫痫	1=是 2=否				
34.22	甲状腺类疾病	1=是 2=否				
34.23	其他，请说明__________	1=是 2=否				
34.24	其他，请说明__________	1=是 2=否				
34.25	其他，请说明__________	1=是 2=否				
34.26	其他，请说明__________	1=是 2=否				
34.27	其他，请说明__________	1=是 2=否				
34.28	其他，请说明__________	1=是 2=否				

			第一次	第二次	第三次	第四次
35.	这次培训有多少个课时？	个				
36.	这次培训的每个课时多长时间？	分钟				
37.	这次培训的费用（没有写 0），不包括交通食宿费用	元				
38.	这次培训的交通食宿费用是否主要由您自己承担的？	1=是　　2=否<br>3=无此项费用				
39.	这次培训对提高您的医疗技能是否有帮助？	1=完全没有帮助 2=基本没有帮助<br>3=有一些帮助　 4=比较有帮助<br>5=非常有帮助				

	问 题	单位/选项	答 案
40.	如果有机会，您最想接受哪个方面的远程在线培训？**（单选）（必须让医生知晓所有选项）**	0=什么培训都不想接受<u>>>**42 题**</u><br>1=高血压 13=急诊与急救<br>2=糖尿病 14=妇科疾病<br>3=肺结核 15=男科疾病<br>4=艾滋病 16=骨科疾病（如骨折与骨关节炎）<br>5=精神障碍或精神病 17=皮肤病<br>6=慢性肺病 18=外科急腹症<br>7=冠心病 19=护理<br>8=小儿腹泻 20=鼻炎<br>9=抗生素的合理使用 21=儿童癫痫<br>10=中草药 22=甲状腺疾病<br>11=中医理疗 23=其他，请说明<br>12=体格检查	
41.	对于最希望接受的远程在线培训，您愿意最多支付多少培训费用	元	
42.	您最愿意参加哪种培训？	1=现场培训<br>2=远程在线培训<br>3=两种培训都想参加<br>4=不想参加任何培训<br>5=其他，请说明	

B. 接诊情况

<u>**指导语：**</u>下面是您在接诊过程中碰到的一些情况。

	问 题	单位/选项	答 案
1.	在您接诊的病人中，通常会有百分之多少的病人需要打针或输液？	%（没有填 0）	
2.	您觉得医生有百分之多少的责任要确保病人按照医嘱服药？（不进行解释）	%（没有填 0）	
3.	在您接诊的病人中，您估计有百分之多少的病人不会按照医嘱服药？	%（没有填 0）	
4.	在您接诊的病人中，您估计有百分之多少的病人会直接要求您给开抗生素？	%（没有填 0）	
5.	在您接诊的病人中，您估计有百分之多少的病人，没有明确要求但您感觉他们希望您给开抗生素？	%（没有填 0）	
6.	如果一个病人家里比较穷，您会给他如何开药？<br>*（注意：这道题不要读选项）*	1=根据经济情况开<br>2=根据病情情况开<br>3=综合考虑	
7.	当病人走进诊所，只通过观察病人而不进行任何问诊和检查，您有百分之多少的把握能知道病人得的是什么病？（不进行解释）	%（没有填 0）	

C.常见症状治疗情况

指导语：接下来是我们想了解过去1个月，您碰到的有如下症状的患者的情况。

调查员须知：如果在回答 b 题时，医生说不给开抗生素，调查员就和医生强调说“不是问真实的情况，而是您估计通过吃抗生素可以治好的可能性”；即使该部分 a 题医生回答为 0，b 题也需要继续问。

症 状		a. 过去 1 个月，您接诊了多少人次有该症状的病人？（人次，没有填 0）	b.您估计该症状的一个病人通过吃抗生素可以治好的可能性有百分之多少？（%）
1.	拉肚子		
2.	呼吸困难		
3.	同时头疼，脸有些发烫		
4.	同时流鼻涕，咳嗽，全身无力		

D.考核情况

指导语：接下来想了解一下2016年上级机构对您的考核情况，包括考核时间、考核内容以及相应的奖惩措施等。

问 题		单位/选项	答 案
1.	2016 年，上级机构是否对您进行考核？	1=是 2=否 **>>E 部分**	
2.	多长时间考核一次？	1=一年一次 2=半年一次<br>3=一季度一次 4=每月一次<br>5=每周一次 6=其他，请说明	
3.	2016 年，上级机构是否对您承担的公共卫生服务进行考核？	1=是 2=否	
4.	2016 年，上级机构是否对您的药品处方进行考核？	1=是 2=否	
5.	2016 年，上级机构是否对您抗生素的合理使用进行考核？	1=是 2=否	
6.	如果考核结果优秀，您会不会得到奖金？	1=会 2=不会	
7.	如果考核结果不合格，您会不会被扣钱？	1=会 2=不会	

E.收入

指导语：接下来想了解一下您大概的收入

问 题		单位/选项	答 案
1.	2016 年，您当医生的基本工资，基本工资是指上级机构给村医发的固定工资，和工作量不相关、不需要考核的是多少？	元/年（没有填 0）	
2.	在 2016 年村诊所的纯收入中，分给您的占多大比例？（如果该诊所不止一个人并且调查了所有人，那么诊所内被调查的人员回答的这一题的答案加起来必须等于 100%）	%	
3.	除了当医生外，您还有其他工作吗？	1=有 2=没有>> **F 部分**	
4.	您还有哪些其他工作？**（念完所有选项；可多选，选项之间用逗号隔开）**	1=务农但家里所有农产品仅自给自足（调查员不需要再问此部分收入）<br>2=务农并有农产品出售<br>3=打零工 4=做生意<br>5=村干部 6=其他，请说明	
5.	2016 年除了在本村诊所当村医的收入，您个人其他的收入有多少？（比如说种地、打零工或者医生在前面提到的其他事情）	元	

F. 手机与网络

指导语：接下来了解您平时获取信息的相关情况。

问题		单位/选项	答案
1.	您有没有智能手机（安卓/苹果手机）或者电脑（包括办公电脑和家里的电脑）？	1=都有 2=只有智能手机<br>3=只有电脑 4=都没有>> **11 题**	
2.	如果有，该手机或者电脑能上网吗？	1=能 2=不能>>**11 题**	
3.	本人是否会操作电脑？	1=是 2=否	
4.	您在手机或者电脑上用 QQ 吗？	1=用 2=不用	
5.	您在手机或者电脑上用微信吗？	1=用 2=不用	
6.	您经常在手机/电脑上学习或查找医疗知识吗？	1=一周 1 次或多次<br>2=一月 1-3 次<br>3=半年 1-5 次<br>4=一年不超过 1 次	
7.	你们县或乡镇内的村医之间是否有微信群或 QQ 交流群？	1=都没有>>9 题<br>2=仅乡镇内的村医有<br>3=县内的村医有，乡镇内的没有<br>4=县和乡镇层面都有	
8.	你在上述 QQ 群或者微信群会和其他村医交流医疗知识吗？	1=不交流<br>2=交流，但是只问问题<br>3=交流，只回答问题<br>4=交流，既问问题也回答问题	
9.	你们村医和乡镇卫生院和县医院的医生之间是否有微信群或 QQ 交流群？	1=都没有>>11 题<br>2=仅和乡镇卫生院之间有<br>3=仅和县医院的医生有<br>4=都有	
10.	你在上述 QQ 群或者微信群会和乡镇卫生院和县医院的医生交流医疗知识吗？	1=不交流<br>2=交流，但是只问问题<br>3=交流，只回答问题<br>4=交流，既问问题也回答问题	
11.	您遇到不知如何处理的病症，主要从何种途径获取答案？（选项必须全部读完）	1=QQ 群或微信群内咨询<br>2=打电话求助认识的医生<br>3=自己在手机或电脑上上网查询<br>4=查找医学书<br>5=其他，请说明<br>6=不处理（包括转诊）	

G．抗生素部分

指导语：医生，下面这一部分了解您对抗生素的看法。

调查员须知：从G2部分开始让医生自己选，不需要读，也不给医生做任何解释，但要帮助医生操作平板。

G1.抗生素使用基本情况

问题		单位/选项	答案
1.	在您 2016 年开的所有抗生素药品中，分别有百分之多少是为了以下原因：（**注意：1+2+3+4=100%**）		
	1.1 为了直接用于疾病治疗	%	
	1.2 为了防止现有疾病变成更严重的疾病	%	
	1.3 为了得出对疾病的诊断结果	%	
	1.4 其他原因	%	
2.	您了解如何使用抗生素的信息来源主要是什么？	1=我以往的临床经验<br>2=上级机构发的学习材料/政策文件<br>3=卫生部门的培训<br>4=药品公司的材料<br>5=药物销售员<br>6=其他村医<br>7=课本<br>8=网络	

G2.关于抗生素的一些社会看法，您的观点是什么？

指导语：医生，接下来的部分由您自己来填写，我们不给您做解释。**下列选项都是单选题，请认真读题和作答，选择正确的选项。**

3.	感冒时使用抗生素可以迅速康复	1=正确　2=错误	
4.	在什么情况下，病人应该停止服用抗生素？	1=病人感觉更好时；<br>2=当病人服用完了开的抗生素<br>3=不知道	
5.	对于不确定的病例，最好的办法是将抗生素作为治疗方案的一部分	1=完全同意　2=有点同意<br>3=中立　4=有点不同意<br>5=完全不同意	
6.	为了让病人相信我的医术，即使有时候可能不必要，我也会给病人开抗生素	1=完全同意　2=有点同意<br>3=中立　4=有点不同意<br>5=完全不同意	
7.	病人经常自己直接要求医生给他开抗生素	1=完全同意　2=有点同意<br>3=中立　4=有点不同意<br>5=完全不同意	

8.	当病人非常想要抗生素时，很难不给病人开抗生素，即使我不认为他们需要抗生素。	1=完全同意　2=有点同意<br>3=中立　　　4=有点不同意<br>5=完全不同意	
