# Supplementary material for: Market competition and demand for skills in a credence goods market: Evidence from face-to-face and web-based non-physician clinician training in rural China
Source: PLoS One. 2020 Jun 18;15(6):e0233955. doi: 10.1371/journal.pone.0233955 (PMC7302647; doi:10.1371/journal.pone.0233955)
Supplement: S2 File — (PDF) [file pone.0233955.s002.pdf]

Form code： □□□□□

Village Clinic Facility Form 1 (Year 2017)

City： ______________________________

County（District）： ______________________________

Town： ______________________________

Village： ______________________________

Name of village clinic： ______________________________

Name of director： ______________________________

Contact number of director： _ _ _ _ _ _ _ _ _ _ _

Name of interviewee： ______________________________

Contact number of interviewee： _ _ _ _ _ _ _ _ _ _ _

Date of interview：2017/_____(MM)____(DD) Interviewer：_______________

Start time of interview:___(Hour)___(Minute) End time of interview:___(Hour)___(Minute)

Dear doctor：hello！

Thank you very much for participating in the survey of "Yunnan Health Service Research". The project is funded by "Health and Hope Fund" of the Business Development Center of the Red Cross Society of China (RCSC) (Beijing) and UCB (Belgium). We are research team from Peking University. The project aims to provide policy recommendations for improving rural healthcare service by understanding the status of primary healthcare in the rural area. Your support and cooperation are very important to us. We guarantee that your answers will only be used for research purposes and kept strictly confidential. Please do not worry. Thank you for your cooperation.

Notice for enumerator：

1）Read questions and options completely. Please write the doctor's answers in the answer column. Unless otherwise specified, questions refer to the situation in 2017 (this year).

2）If a question requires number, please write the specific number instead of a range. If a doctor is used to answering a range, tell him /her "We need a specific number instead of a range. Please try to answer a specific number". If the doctor still cannot give a specific number after 3 reminds, you should take the average of the range.

3）If the doctor does not know how to answer, write 888 and his / her reason for not knowing how to answer. If there is a special case and you don't know how to write, write 999 and the special case.

4）You do not need to read the texts in the bracket. Read them to the doctor if he or she has a question.

A. List of clinic staff

Introduction: Next we want to know about the staff in the clinic.

a．Name of the staff		b. Gender<br>1=Male<br>2=Female	c. Age（years old）	d．Responsibility<br>1=Consultation and prescription<br>2=Consultation and prescription but only on Chinese herbal medicine >>Next staff<br>3=Not for consultation and prescription >>Next staff	e. Did he / she answer the doctor form?（If the staff has not been working in the clinic for more than 4 months, then he / she doesn't need to answer the doctor form）<br>1=Yes➔Next staff<br>2=No	Notes (Reasons that he / she didn't answer the doctor form）
1.						
2.						
3.						
4.						
5.						

B. Basic information about the clinic

Introduction： Next we want to know about the basic information about the clinic.

	Question	Unit / Options	Answer
1.	How many of the doctors responsible for consultation and prescription are township hospital formal establishment staff?（The answer should be less than or equal to the sum of "1"s answered for Question d in Part A）	Number of staff<br>**>>If 0，then go to Question 3**	
2.	Names of the township hospital formal establishment staff		
3.	Is your clinic a designated medical institution of NCMS?	1=Yes<br>2=No>> Part C	
4.	Can the villagers who are participants of NCMS get reimbursed from NCMS for the cost of visits to your clinic?	1=Yes 2=No	

C. Service

C1. Basic information

Introduction: Next we want to know about the basic information on the clinic service.

	Question	Unit / Options	Answer
1.	Including your clinic, how many clinics are there within 5 km of your clinic as long as one can see a doctor there?	Number of clinics	
2.	How many pharmacies are there within 5 km of your clinic, including drugstores and excluding clinics?（Answer 0 if none）	Number of pharmacies	
3.	How many permanent residents are there within 5 km of your clinic, including local residents and migrants?	Number of person	
4.	How many of the permanent residents population within 5 km of your clinic visit your clinic?	%	
5.	How many patients visited your clinic in the last month?	Number of visits	
6.	Among them, how many did you recommend to be referred to a superior hospital?（6<5）	Number of visits	
7.	How many patients whom you didn't know visited your clinic in the last month?	Number of person	
8.	Does your clinic charge a general consultation fee, including that being exempted or deducted for participants of NCMS?	1=Yes, we charge for all patients<br>2= Yes, we charge for some patients（Please elaborate）<br>3=No **>>Question 10**	
9.	If yes, how much is the general consultation fee? (Check if the answer is the amount after exemption deduction)	Yuan/visit	
10.	Does your clinic use intramuscular injection?	1=Yes 2=No **>> Question 12**	
11.	How many patients received intramuscular injection in the last month? (11<5)	Number of visits	
12.	Does your clinic use intravenous injection?	1=Yes 2=No **>>Question 14**	
13.	How many patients received intravenous injection in the last month?？(13<5)	Number of visits	
14.	Did your clinic provide public health service in 2016?	1=Yes 2=No **>> Part C2**	
15.	How many administrative villages were your clinic responsible for the public health service in 2016?	Number of villages（allow decimals，such as 0.5）	
16.	What is the population of the administrative villages mentioned above?	Number of person	

C2.Medical equipment and use

<u>Introduction：</u> Next we want to know about the medical equipment and their use. Please show me the equipment when I asked about a specific equipment.

Notice for enumerator： Note all equipment is to be seen as true. Enumerator should confirm with the doctor that the equipment is available for use. An equipment cannot be counted if it breaks down and the doctor has no plan to fix it.

**Name of the equipment**	**a．Do you have this equipment?<br>1=Yes<br>2=No<u>>>Next line</u>**	**b．Is it distributed by upper-level authorities for free?<br>1=Yes<br>2=No**	**c．Was it used in the last week?<br>1=Yes<br>2=No**
1. Stethoscope			
2. Thermometer			
3. Sphygmomanometer			
4. Tongue spatula			
5. Tourniquet			
6. Treatment disc			
7. Visiting box			
8. High temperature sterilizer with pressure gauge			
9. UV disinfection lamp			
10. Debridement suture package			
11. Hospital bed			
12. Sputum suction			
13. Refrigerated bag			
14. Oxygen bag or bottle			
15. Blood routine tester			
16. Blood glucose monitor			
17. Height and weight meter			
18. Trash can with lid			
19. Computer			
20. Printer			
21. Medicine cabinet			
22. Vacuum cupping			
23. Acupoint therapy instrument			
24. Moxibustion box			
25. Massage bed			
26. Multi-parameter health tester			
27. Freezer or refrigerator			
28. Specific electromagnetic wave treatment instrument or magic lamp			

29. Other 1, please specify_____			
30. Other 2, please specify ____			
31. Other 3, please specify ____			
32. Other 4, please specify ____			
33. Other 5, please specify ____			
34. Other 6, please specify ____			
35. Other 7, please specify ____			
36. Other 8, please specify ____			

D. Visit records

Introduction: Next we want to know about the visit records kept in your clinic. Please take out all the documents related to patient visits so that we can look at it together.

Notice for enumerator: For Questions 1 to 11, ask the doctor what records and documents are related to patient visits and ask him / her to take them out. Answer the questions for all kinds of records. Records specific to a few special patients are not counted (for example, only keeping the record for patients who owe money).

	Question	Unit / Options	Answer
1.	Do you keep records for patient visits, such as patient information, outpatient logs and prescriptions?（If only keeping records on name of patient and visit date, then it is not counted and choose 2=No for this question）	1=Yes<br>2=No **>>Question 8**	
2.	In what form is the record kept?	1=Paper records<br>2=Electronic records 3=Both	
3.	Dose the record include personal information of patients? (Name, gender, age, etc.)	1=Yes 2=No	
4.	Does the record include a patient's main symptoms?	1=Yes 2=No	
5.	Does the record include a patient's past medical history?	1=Yes 2=No	
6.	Does the record include the diagnosis of a patient?	1=Yes 2=No	
7.	Does the record include the treatment plan (prescription) for a patient?	1=Yes 2=No	
8.	Did upper-level authorities inspect the medical records or prescription kept in the clinic in 2016?	1=Yes 2=No **>>Part E**	
9.	How often were you inspected in 2016?	1=In three months<br>2=In three to six months<br>3=In six to nine months<br>4=In nine months to a year<br>5=More than a year	
10.	Would there be a monetary penalty if an obvious error was found?	1=Yes 2=No	
11.	Would there be an administrative penalty if an obvious error was found, such as verbal criticism and notice of criticism?	1=Yes 2=No>>Part **E**	
12.	What administrative penalties will be imposed if an obvious error was found? (Multiple choices, read all the options)	1=Verbal criticism<br>2=Notice of criticism<br>3=Revocation of Village Doctor Practice Certificate<br>4=Revocation of Village Clinic Business License<br>5=Other, please specify	

E. Medicine and use

Introduction: Next we want to know about the medicine that your clinic has and their use.

Question		Unit / Options	Answer
1.	By the end of 2016, how many kinds of western medicines did your clinic have?	Number of kinds	
2.	By the end of 2016, how many kinds of Chinese patent medicines did your clinic have?	Number of kinds	
3.	By the end of 2016, how many kinds of Chinese herbal medicines did your clinic have?	Number of kinds	
4.	By the end of 2016, what was the total value of the medicines that your clinic had?	Yuan	
5.	By the end of 2016, how much of the medicines was purchased by your clinic based on the kinds of medicines? Excluding the medicines purchased by National Basic Medicine Platform and township hospitals.	%（Answer 0 if none）	
6.	By the end of 2016, how much of the medicines was purchased by your clinic based on purchasing value? Excluding the medicines purchased by National Basic Medicine Platform and township hospitals.	%（Answer 0 if the answer for Question 5 is 0）	
7.	Did your clinic implement the zero-difference rate policy? (That's the sale price of medicine equals to its purchase price)	1=Yes<br>2=No**>> Part F**	
8.	Did upper-level authorities evaluate the implementation of the zero-difference rate policy of your clinic in 2016?	1=Yes<br>2=No	

E. Location of the clinic

Introduction: Last, we want to know about the geographic location and economy of the village.

Question		Unit / Options	Answer
1.	How far is the clinic from the township hospital?	Km	
2.	Is there a shuttle bus or van from the village to the township hospital operating every day?	1=Yes<br>2=No>>Question 4	
3.	How much does a one way trip cost if a patient takes a shuttle bus or van from the village to the township hospital (renting a private car is not counted)?（The largest amount）	Yuan	
4.	How far is the clinic from the county hospital that villagers visit the most frequently?	Km	
5.	Is there a shuttle bus or van from the village to the county hospital that villagers visit the most frequently operating every day?	1=Yes<br>2=No>>Question 7	
6.	How much does a one way trip cost if a patient takes a shuttle bus or van from the village to that county hospital (renting a private car is not counted)?（The largest amount）	Yuan	
7.	What is the average daily wage for a 50-year-old male who works on odd jobs (not a skilled worker)?	Yuan	

8.	What is the average daily wage for a 50-year-old female who works on odd jobs (not a skilled worker)?	Yuan	

Form filled by（Signature） ___________

Form checked by（Signature） ___________

Form code：□□□□□

Village Clinic Facility Form 2 (Year 2017)

A. Common diseases management

Introduction： Next, we want to know your how your clinic manage common diseases.

Notice for enumerator： Ask the questions by column. Insert the diseases into the questions when asking.

	tuberculosis	Type 2 diabetes	hypertension	Pharyngitis	Pediatric diarrhea	Asthma	Chronic lung disease	Angina pectoris
1.Was your clinic responsible for then management of patients with XX in 2016?（1=Yes 2=No>>**Question 3**）								
2.How many patients with XX you're your clinic manage in 2016?（Answer 0 if none）								
3. How many suspected cases of XX did your clinic find in 2016?【Go to next disease if none】								
4.Among them, how many did you recommend referral (including verbal referral) no matter patient did go or not?								
5.Among them, how many did you reported to upper-level authorities?								

B. Income of 2016

Introduction：Next we want to know about the income of 2016. Let's calculate.

Notice for enumerator： If the doctor's answers to Question 5 and 6 in Village Clinic Facility Form 1 are not zero, which means the medicines that the doctor purchased are not zero, then there must exists net income from non-zero rate medicines. (If there is a hired doctor, answer to Question 7 should include the wages for him/her. The wage is included in the net income of the clinic and the clinic distributes its total income.)

Questions		Unit / Options	Answer
1.	Subsidy for public health service	Yuan	
2.	Subsidy for implementation of the zero-mark-up policy	Yuan	
3.	Total of the fixed salaries that all the staff received from county health department	Yuan	

4.	Net income from medication, excluding medicines.	Yuan	
**5.**	Net income from non-zero-mark-up medicines	Yuan	
**6.**	Other income（the income is not counted if it is from doctor's farming or other things irrelevant to the clinic, please specify_____	Yuan	
7.	Total income of the clinic<br>（First ask the total income of the clinic. Then compare the amount with the summation of the answers for Question 1 to 6. If the amounts are inconsistent, ask for the reasons. 7=1+2+3+4+5+6）	Yuan	

Form code: □□□□□□

Village Clinic Doctor Form (Year 2017)

City: ______________________________

County（District）: ______________________________

Town: ______________________________

Village: ______________________________

Name of village clinic: ______________________________

Name of village doctor: ______________________________

Contact number of village doctor: _ _ _ _ _ _ _ _ _ _ _

Date of interview：2017/_____(MM)____(DD)　　Interviewer：_______________

Start time of interview:___(Hour)___(Minute)　　End time of interview:___(Hour)___(Minute)

Introduction：Next，We will start filling in the doctor form which focuses on all the doctors responsible for inquiry or prescription (the number of which equals to the total number of "1"answered in the Column e of Part A in the Village Clinic Facility Form).

A. Basic information about doctor

A1. Personal information

Introduction: First, please answer the questions regarding your personal information.

Question		Unit / Options	Answer
1.	Gender	1=Male 2=Female	
2.	Ethnic	1=Han 2=Yi<br>3=Bai 4=Hani<br>5=Zhuang 6=Miao<br>7=Hui 8=Lisu<br>9= Lahu 10=Wa<br>11=Naxi 12=Yao<br>13=Other, please specify_______	
3.	Birth Date	Example：19670120	
4.	Are you native in the village?	1=Yes 2=No	
5.	What's your responsibility in the clinic?	1=All the work<br>2=Medication<br>3=Public health<br>4= Other, please specify	
6.	When did you first start being a doctor?	Year, such as 1988	
7.	Since then, how many years did you work on other jobs instead of being a doctor（Part-time jobs are not taken into account）?	Years（answer 0 if never worked on other jobs；can be decimal）	
8.	Since when have you been working in this clinic（note not be earlier than the birth year）	Year, such as 1997	
9.	What's your main reason of being a doctor?（Must read through all the options）	1=Having a relative or family member being a doctor<br>2=Learned medicine before<br>3=There was no doctor in the village<br>4=Enjoying being a doctor<br>5=To make a living<br>6=Elected by the village collective<br>7=Other, please specify	
10.	Do you have any family member or relative being a village doctor before your practice?	1=Yes 2=No	
11.	Do you wish your children or grandchildren to be village doctors in the future?	1=Yes 2=No	
12.	In 2016, of the time for doctor-related work, what percentage was devoted to public health service? (Less than 100)	%	

13.	In the last week, how many hours did you spend on doctor-related work? Deduct the time for meals, sleep, leisure and other things.	Hours（Less than 168 hours）	

A2. Education

Question		Unit / Options	Answer
1.	What is your highest education level, including adult education?（check the certificate）	0=None<br>1=Elementary school or below（including failed to graduate from junior high school）<br>2=Junior high school（including failed to graduate from senior high school）<br>3=Senior high school<br>4=Vocational high school<br>5=Junior college<br>6=College<br>7=Graduate school or above	
2.	What is your highest achieved qualification certificate?（check the certificate）	0=None<br>1=Village doctor qualification certificate<br>2=Practicing assistant physician<br>3=Practicing physician<br>4=Other, please specify	
3.	Have you received any diploma medical education, including full-time education, in-service education and correspondence education? (check the certificate)	1=Yes<br>2=No >> **Question 9**	

Introduction: Next we want to know about each of your diploma medical education experience, which includes full-time education， in-service education and correspondence education, but excludes training.【Better to check the education certificate to make sure it is diploma education】

Notice for enumerator： Question 4 to 8 are dependent on the answer to Question 3. Read each question completely.

Question		Unit / Options	Medical Education			
			The first time	The second time	The third time	The fourth time
4.	The year completed the education	Year（such as 1990）				
5.	Is it full-time education, which means studying at school without any work?	1=Yes 2=No				
6.	What is the education level? **（Notice：if the doctor is not sure, enumerator should decide according to the certificate）**	1=Vocational high school<br>2=Junior college<br>3=College<br>4=Graduate school and above				
7.	What is the major?	1=Chinese medicine<br>2=Western medicine<br>3=Combination of Chinese and western medicine<br>4=Public health<br>5=Nursing<br>6=No specific major				

		7=Other, please specify				
8.	Did you pay for the education fully?	1=Yes 2=No				
9.	Have you received any non-diploma medical training before being a doctor?	1=Yes 2=No **>> Part A3**				
10.	How many days of non-diploma medical training did you receive before being a doctor?	Days				

A3. Training

Introduction: Next we want to know about the face-to-face medical training that you participated in 2016.

	Question	Options	Answer				
1.	Did you participate in medical trainings that were organized by township hospitals in 2016?	1=Yes<br>2=No **>> Question 4**					
2.	If yes, how many times did you participate in 2016?	Times					
3.	What were the main contents of the trainings organized by township hospitals in 2016?	1=Emphasis on medication<br>2=Emphasis on public health<br>3=Emphasis on both medication and public health<br>4=Other, please specify					
4.	Did you participate in any face-to-face medical training organized by other institutes other than township hospitals in 2016? (Excluding web-based training)	1=Yes<br>2=No **>> Question 23**					
	Question	Options	Answer				
			a. County health department	b. Municipal health department	c. Other institute 1:________	d. Other institute 2: ________	e. Other institute 3:______
5.	Have you participated in medical trainings organized by XXX (ask in the order of county health department, municipal health department and other institutes) in 2016?	1=Yes<br>2=No >>**Next column**					
6.	If yes, how many times did you participate in the medical trainings organized by the institute in 2016?	Number of times					

7.	For the Xth training, what is X?	X	County health department X	Municipal health department X	Other institute 1:_______X	Other institute 2: _____ X	Other institute 3:______ X
8.	Which month did the training start on?	Month（such as 7）					
9.	How long did the training take, excluding commuting time?	Days（allow decimals）					
9.1.	How long did the training actually last, excluding commuting time? (9.1<=9)	Days（allow decimals）					
10.	Did the training include clinical practice?	1=Yes 2=No					
11.	Was the training free?	1=Yes **>>Question 13**<br>2=No					
12.	If not, how much was the fee?	Yuan					
13.	Did you **mainly** afford the transportation and accommodation fee caused by the training?	1=Yes<br>2=No **>> Question 15**					
14.	If yes, how much was the transportation and accommodation fee	Yuan					
15.	Did your clinic operate normally during this training?	1=Yes **>>Question 17**<br>2=No					
16.	How much was it expected to earn during this training if your clinic had operated normally?	Yuan					
17.	How many times did you consult an expert within the month after the training?	Number of times（answer 0 if none）					
18.	Do you think the training was helpful for improving medical techniques?	1=Not helpful at all<br>2=Basically not helpful<br>3=A little helpful<br>4=Helpful<br>5=Very helpful					

19.	How much were you willing to pay for the training fee if it was not free?	Yuan					
20.	How much were you willing to pay for the transportation and accommodation fee for this training?	Yuan					
21.	Where did the trainers mainly come from?	1=Doctors from village or community<br>2=Doctors from township or street office<br>3=Doctors from county hospitals<br>4=Doctors from municipal hospitals<br>5= Doctors from provincial hospitals<br>6=Other, please specify					
22.	What were the contents of the training?						
22.1	Hypertension	1=Yes 2=No					
22.2	Diabetes	1=Yes 2=No					
22.3	Tuberculosis	1=Yes 2=No					
22.4	AIDS	1=Yes 2=No					
22.5	Mental disorder or mental illnesses	1=Yes 2=No					
22.6	Chronic lung diseases	1=Yes 2=No					
22.7	Coronary heart diseases	1=Yes 2=No					
22.8	Pediatric diarrhea	1=Yes 2=No					
22.9	Proper use of antibiotics	1=Yes 2=No					
22.10	Chinese herbal medicine	1=Yes 2=No					
22.11	TCM physiotherapy	1=Yes 2=No					
22.12	Physical examination	1=Yes 2=No					
22.13	Emergency and first aid	1=Yes 2=No					
22.14	Gynecological diseases	1=Yes 2=No					
22.15	Andrology	1=Yes 2=No					

22.16	Orthopedic diseases（such as fractures and osteoarthritis）	1=Yes 2=No					
22.17	Skin diseases	1=Yes 2=No					
22.18	Surgical abdomen	1=Yes 2=No					
22.19	Nursing	1=Yes 2=No					
22.20	Rhinitis	1=Yes 2=No					
22.21	Child epilepsy	1=Yes 2=No					
22.22	Thyroid diseases	1=Yes 2=No					
22.23	Other, please specify___________	1=Yes 2=No					
22.24	Other, please specify___________	1=Yes 2=No					
22.25	Other, please specify___________	1=Yes 2=No					
22.26	Other, please specify___________	1=Yes 2=No					
22.27	Other, please specify___________	1=Yes 2=No					
22.28	Other, please specify___________	1=Yes 2=No					

Question		Unit / Options	Answer
23.	If possible, what kind of training do you want most?（Excluding web-based training）？ **（Single choice）** **（Let the doctor know all the options）**	0=None **>> Question 28**<br>1= Hypertension<br>2= Diabetes<br>3= Tuberculosis<br>4= AIDS<br>5= Mental disorder or mental illnesses<br>6= Chronic lung diseases<br>7= Coronary heart diseases<br>8= Pediatric diarrhea<br>9= Proper use of antibiotics<br>10= Chinese herbal medicine<br>11= TCM physiotherapy<br>12= Physical examination<br>13= Emergency and first aid<br>14= Gynecological diseases<br>15= Andrology<br>16= Orthopedic diseases（such as fractures and osteoarthritis）<br>17= Skin diseases<br>18= Surgical abdomen<br>19= Nursing<br>20= Rhinitis<br>21= Child epilepsy<br>22= Thyroid diseases<br>23= Other, please specify	
24.	For your most wanted training, in what way of delivery do you most prefer? **（Single choice）（Let the doctor know all the options）**	1=Seminar lecture<br>2=Interactive teaching<br>3=Scenario simulation<br>4=Clinic practice<br>5=Other, please specify	
25.	For your most wanted training, how many days at most are you willing to spend on it off-job?	Days（allow decimals）	
26.	For your most wanted training, how much at most are you willing to pay? Only for the training fee, not including the transportation and accommodation fee.	Yuan	
27.	For your most wanted training, are you willing to pay for the transportation and accommodation fee?	1=Yes 2=No	
28.	Did you participate in any training at the county level or above from 2013 to 2015?	1=Yes 2=No	
29.	Did you take any internship at a upper-level hospital from 2013 to 2016?（Excluding trainings mentioned above）？	1=Yes 2=No	

Distance training

Introduction： Next we want to know about the distance trainings that you participated in 2016.

Question		Unit / Options	Answer
30.	Did you participate in any form of distance online training that is related to your clinical work in 2016?	1=Yes<br>2=No **>>Question 40**	
31.	If yes, how many times did you participate in?	Number of times	

			The first time	The second time	The third time	The fourth time
32.	How did you obtain the opportunity to participate in this training?	1=Upper-level authorities 2=Other doctors 3=The Internet 4=Other, please specify				
33.	Where did you participate in this training?	1=Home / clinic 2=Village committee office 3=Township hospital 4=Other, please specify				
34.	What are the contents of the training?					
34.1	Hypertension	1=Yes 2=No				
34.2	Diabetes	1=Yes 2=No				
34.3	Tuberculosis	1=Yes 2=No				
34.4	AIDS	1=Yes 2=No				
34.5	Mental disorder or mental illnesses	1=Yes 2=No				
34.6	Chronic lung diseases	1=Yes 2=No				
34.7	Coronary heart diseases	1=Yes 2=No				
34.8	Pediatric diarrhea	1=Yes 2=No				
34.9	Proper use of antibiotics	1=Yes 2=No				
34.10	Chinese herbal medicine	1=Yes 2=No				
34.11	TCM physiotherapy	1=Yes 2=No				
34.12	Physical examination	1=Yes 2=No				
34.13	Emergency and first aid	1=Yes 2=No				
34.14	Gynecological diseases	1=Yes 2=No				
34.15	Andrology	1=Yes 2=No				
34.16	Orthopedic diseases（such as fractures and osteoarthritis）	1=Yes 2=No				
34.17	Skin diseases	1=Yes 2=No				
34.18	Surgical abdomen	1=Yes 2=No				
34.19	Nursing	1=Yes 2=No				
34.20	Rhinitis	1=Yes 2=No				
34.21	Child epilepsy	1=Yes 2=No				
34.22	Thyroid diseases	1=Yes 2=No				
34.23	Other, please specify___________	1=Yes 2=No				
34.24	Other, please specify___________	1=Yes 2=No				
34.25	Other, please specify___________	1=Yes 2=No				
34.26	Other, please specify___________	1=Yes 2=No				
34.27	Other, please specify___________	1=Yes 2=No				
34.28	Other, please specify___________	1=Yes 2=No				
			The first time	The second time	The third time	The fourth time

35.	How many training hours did the training have?	Number of training hours				
36.	How many hours did one training hour have?	Minutes				
37.	Training fee（answer 0 if none）, excluding transportation and accommodation fee	Yuan				
38.	Did you mainly afford the transportation and accommodation fee for this training?	1=Yes 2=No<br>3=No transportation and accommodation fee				
39.	Do you think the training was helpful for improving medical techniques?	1=Not helpful at all<br>2=Basically not helpful<br>3=A little helpful<br>4=Helpful<br>5=Very helpful				

	Question	Unit / Options	Answer
40.	If possible, what kind of online training do you want most? **（Single choice）** **（Let the doctor know all the options）**	0=None **>> Question 42**<br>1= Hypertension<br>2= Diabetes<br>3= Tuberculosis<br>4= AIDS<br>5= Mental disorder or mental illnesses<br>6= Chronic lung diseases<br>7= Coronary heart diseases<br>8= Pediatric diarrhea<br>9= Proper use of antibiotics<br>10= Chinese herbal medicine<br>11= TCM physiotherapy<br>12= Physical examination<br>13= Emergency and first aid<br>14= Gynecological diseases<br>15= Andrology<br>16= Orthopedic diseases（such as fractures and osteoarthritis）<br>17= Skin diseases<br>18= Surgical abdomen<br>19= Nursing<br>20= Rhinitis<br>21= Child epilepsy<br>22= Thyroid diseases<br>23= Other, please specify	
41.	For your most wanted online training, how much at most are you willing to pay?	Yuan	
42.	Which mode of training do you want most?	1=Face-to-face training<br>2= Web-based training<br>3=Both<br>4=Neither<br>5=Other, please specify	

B. Admissions

Introduction： The following are some situations during an admission.

Question		Unit / Options	Answer
1.	What is the percentage of your patients who need injection or intravenous infusion?	%（answer 0 if none）	
2.	What is the percentage of responsibility for doctor to make sure patients to take the medicine according to medical advice?（Do not explain）	%（answer 0 if none）	
3.	Basic on your estimation, what is the percentage of your patients who do not take the medicine according to medical advice?	%（answer 0 if none）	
4.	Basic on your estimation, what is the percentage of your patients who ask you to prescribe antibiotics directly?	%（answer 0 if none）	
5.	Basic on your estimation, what is the percentage of your patients who want you to prescribe antibiotics but did not ask so directly?	%（answer 0 if none）	
6.	How would you prescribe if a patient is poor? *（Notice：do not read the options）*	1=Prescribing based on economic condition<br>2=Prescribing based on medical condition<br>3=Taking both conditions into consideration	
7.	When a patient walk in, what is the percentage of certainty that you can conclude his/her disease without any inquiry or inspection? （Do not explain）	%（answer 0 if none）	

C. Treatment for common symptoms

Introduction： Next we want to know about if you have had patients with the following symptoms during the last months?

Notice for enumerator：if the doctor says he/she don't prescribe antibiotics when answering Question b, enumerator should emphasize that "We are not asking the real situation but your estimation on the possibility of curing a patient by taking antibiotics". The doctor need to answer Question b even if his/her answer to Question a is 0.

Symptom		a. How many patients had such symptom during the last month?（Number of visits，answer 0 if none）	b. Based on your estimation, what is the percentage of possibility to cure a patient with such symptom by taking antibiotics?（%）
1.	Diarrhea		
2.	Difficult to breathe		
3.	Having a headache and a hot face		
4.	Running at the nose, coughing and feeling of malaise		

D. Assessment

introduction：Next we want to know about the assessments that were conducted by the upper-level authorities in 2016, including time, contents, rewards and punishments.

Question		Unit / Options	Answer
1.	Did upper-level authorities assess you in 2016?	1=Yes 2=**No >>Part E**	
2.	How often is the assessment?	1=Once a year 2=Once a half year<br>3=Once a season 4=Once a month<br>5=Once a week<br>6=Other, please specify	
3.	Did upper-level authorities assess you on public health service in 2016?	1=Yes 2=No	
4.	Did upper-level authorities assess you on prescription in 2016?	1=Yes 2=No	
5.	Did upper-evel authorities assess you on proper use of antibiotics in 2016?	1=Yes 2=No	
6.	Will you receive monetary rewards if the assessment result is good?	1=Yes 2=No	
7.	Will your money be deducted if you fail to pass the assessments?	1=Yes 2=No	

E. Income

Introduction： Next we want to know about your income

Question		Unit / Options	Answer
1.	What was your basic salary in 2016? Basic salary means the fixed salary paid by upper-level authorities, which is irrelevant to the workload and assessments.	Yuan/Year（answer 0 if none）	
2.	How much of the net income of the village clinic was distributed to you in 2016?<br>（If the village clinic has more than one staff and all of them are interviewed, the answers from them should add up to 100%.）	%	
3.	Do you have any other job apart from being a doctor?	1=Yes 2=No>> **Part F**	

4.	What other jobs do you have?<br>（**Read all the options; multiple choices, use comma to separate the answers**）	1=Farming but all the agricultural products are for family consumption（no need to ask income from this）<br>2=Farming and selling agricultural products<br>3=Doing part-time jobs<br>4=Doing business<br>5=Village cadre<br>6=Other, please specify	
5.	What was your income from other jobs apart from that from the clinic in 2016?（For example: farming, doing part-time jobs or other jobs mentioned above.）	Yuan	

F. Phone and the Internet

Introduction：Next we want to know about your source of information.

Question		Unit / Options	Answer
1.	Do you have a smart phone (Android/iPhone) or a computer (including computer at workplace or at home)？	1=Both 2=Only the smart phone<br>3=Only the computer<br>4=Neither>> **Question 11**	
2.	If yes, is it connected to the Internet?	1=Yes 2=No>> **Question 11**	
3.	Do you know how to use computer?	1=Yes 2=No	
4.	Do you use QQ on cell phone or computer?	1=Yes 2=No	
5.	Do you use WeChat on cell phone or computer?	1=Yes 2=No	
6.	How often do you search for medical information on your phone or computer?	1=One or more times a week<br>2=One to three times a month<br>3=One to five times every half year<br>4=No more than once a year	
7.	Do the village doctors in your county or town have a WeChat or QQ group?	1=Neither>> Question 9<br>2=Only among the doctors in the town<br>3=Only among the doctors in the county4=Both	
8.	Do you discuss medical knowledge with other village doctors in the group mentioned above?	1=No<br>2=Yes, but only asking questions<br>3= Yes, but only answering questions<br>4=Yes, both asking and answering questions	
9.	Do the village doctors have a WeChat or QQ group with doctors from township hospitals or county hospitals?	1=Neither>> Question 11<br>2=Only with the doctors from township hospitals<br>3=Only with the doctors from county hospitals<br>4=Both	

10.	Do you discuss medical knowledge with doctors from township hospitals or county hospitals in the group mentioned above?	1=No<br>2=Yes, but only asking questions<br>3= Yes, but only answering questions<br>4=Yes, both asking and answering questions	
11.	If encountering a symptom that you don’t know how to treat, what is your major way to get an answer?（Read all the options）	1=Enquiring in WeChat or QQ group<br>2=Calling an acquainted doctor<br>3=Searching on the Internet by using phone or computer<br>4=Looking up in medicine books<br>5=Other, please specify<br>6=Doing nothing （Including transferring）	

G. Antibiotics

Introduction： Next we want to know about your views on antibiotics

Notice for enumerator： Let the doctor choose the answers in Part G2. Do not read or explain anything. Help the doctor use the iPad.

G1. Use of antibiotics

	Question	Unit / Options	Answer
1.	In all the antibiotics that you prescribed in 2016, what are the percentages of their reasons：（**Notice：1+2+3+4=100%**）		
	1.1 For treating the disease directly	%	
	1.2 For preventing the current disease from exacerbating	%	
	1.3 For getting the diagnosis results of the current disease	%	
	1.4 Other reasons	%	
2.	What is your major source of information on the use of antibiotics?	1= Based on my clinical experience<br>2= Materials / policies given by upper-level authorities<br>3=Trainings by health department<br>4=Materials from pharmaceutical firms<br>5=Salesperson from pharmaceutical firms<br>6=Other village doctors<br>7=Textbooks<br>8=The Internet	

G2. Opinions on antibiotics

Introduction： Next you will answer the questions by yourself. We will not explain the questions or options. **They are all single choice questions. Please read and answer carefully.**

3.	One who has a cold will recover quickly if taking antibiotics.	1=True 2=Wrong	
4.	Under what condition should a patient stop taking antibiotics?	1=When feeling better<br>2=When having taken all prescribed antibiotics<br>3=Do not know	
5.	For those uncertain cases, it's best to use antibiotics as part of the treatment.	1=Completely agree<br>2= Somehow agree<br>3= Neutral<br>4= Somehow disagree<br>5= Completely disagree	
6.	In order to let patients trust on my medical skills, I will prescribe antibiotics even it is unnecessary.	1=Completely agree<br>2= Somehow agree<br>3= Neutral<br>4= Somehow disagree<br>5= Completely disagree	

7.	Patients often ask doctors to prescribe antibiotics.	1=Completely agree<br>2= Somehow agree<br>3= Neutral<br>4= Somehow disagree<br>5= Completely disagree	
8.	It’s hard not to prescribe antibiotics when patients are asking so ,even though I don’t think they need them.	1=Completely agree<br>2= Somehow agree<br>3= Neutral<br>4= Somehow disagree<br>5= Completely disagree	
